# SP-R210 (Myo18A) Isoforms as Intrinsic Modulators of Macrophage Priming and Activation

**DOI:** 10.1371/journal.pone.0126576

**Published:** 2015-05-12

**Authors:** Linlin Yang, Marykate Carrillo, Yuchieh M. Wu, Susan L. DiAngelo, Patricia Silveyra, Todd M. Umstead, E. Scott Halstead, Michael L. Davies, Sanmei Hu, Joanna Floros, Francis X. McCormack, Neil D. Christensen, Zissis C. Chroneos

**Affiliations:** 1 Department of Pediatrics, Pennsylvania State University College of Medicine, Hershey, Pennsylvania, United States of America; 2 Department of Microbiology and Immunology, Pennsylvania State University College of Medicine, Hershey, Pennsylvania, United States of America; 3 Department of Biochemistry and Molecular Biology, Pennsylvania State University College of Medicine, Hershey, Pennsylvania, United States of America; 4 Department of Obstetrics and Gynecology, Pennsylvania State University College of Medicine, Hershey, Pennsylvania, United States of America; 5 Department of Pathology, Pennsylvania State University College of Medicine, Hershey, Pennsylvania, United States of America; 6 Division of Pediatric Critical Care Penn State Hershey Children’s Hospital, Pennsylvania State University College of Medicine, Hershey, Pennsylvania, United States of America; 7 Center of Host Defense and Lung Disease Research, Pennsylvania State University College of Medicine, Hershey, Pennsylvania, United States of America; 8 Pulmonary Immunology and Physiology Laboratory, Pennsylvania State University College of Medicine, Hershey, Pennsylvania, United States of America; 9 The Jake Gittlen Laboratories for Cancer Research, Pennsylvania State University College of Medicine, Hershey, Pennsylvania, United States of America; 10 Department of Internal Medicine, Division of Pulmonary, Critical Care and Sleep Medicine, University of Cincinnati College of Medicine, Cincinnati, Ohio, United States of America; Karolinska Institutet, SWEDEN

## Abstract

The surfactant protein (SP-A) receptor SP-R210 has been shown to increase phagocytosis of SP-A-bound pathogens and to modulate cytokine secretion by immune cells. SP-A plays an important role in pulmonary immunity by enhancing opsonization and clearance of pathogens and by modulating macrophage inflammatory responses. Alternative splicing of the *Myo18A *gene results in two isoforms: SP-R210_S_ and SP-R210_L_, with the latter predominantly expressed in alveolar macrophages. In this study we show that SP-A is required for optimal expression of SP-R210_L_ on alveolar macrophages. Interestingly, pre-treatment with SP-A prepared by different methods either enhances or suppresses responsiveness to LPS, possibly due to differential co-isolation of SP-B or other proteins. We also report that dominant negative disruption of SP-R210_L_ augments expression of receptors including SR-A, CD14, and CD36, and enhances macrophages’ inflammatory response to TLR stimulation. Finally, because SP-A is known to modulate CD14, we used a variety of techniques to investigate how SP-R210 mediates the effect of SP-A on CD14. These studies revealed a novel physical association between SP-R210_S_, CD14, and SR-A leading to an enhanced response to LPS, and found that SP-R210_L_ and SP-R210_S_ regulate internalization of CD14 via distinct macropinocytosis-like mechanisms. Together, our findings support a model in which SP-R210 isoforms differentially regulate trafficking, expression, and activation of innate immune receptors on macrophages.

## Introduction

The surfactant protein A (SP-A) receptor 210 (SP-R210) is a product of the unconventional *Myosin 18A* (*Myo18A*) gene, also known as MysPDZ [1–4], MyoXVIIIA [2, 5, 6], or Myosin-18 [7]. The *Myo18A* gene encodes two SP-R210 splice isoforms in macrophages, namely SP-R210_L_ and SP-R210_S_ [8], also known as MysPDZα or Myo18Aα and MysPDZα or Myo18Aα [1, 9, 10], respectively. In macrophages, SP-R210 mediates phagocytic and immuno-regulatory functions of SP-A [5, 8, 11–17]. The larger SPR210_L_ or Myo18Aα isoforms are distinguished from the short SP-R210_S_ or Myo18Aα isoforms by an amino-terminal extension containing a PDZ domain [3, 5]. In the present report, we use the acronym SP-R210 and Myo18A for immune and non-immune cells, respectively. The reason for this name nomenclature is based on experimental and computational evidence indicating that the *Myo18A* gene is subject to cell type-dependent alternative splicing. For example, in addition to splicing that generates SP-R210_L_ and SP-R210_S_ isoforms, splicing of small exons generates alternate forms of the unique carboxy-terminal domain of Myo18A in macrophages [6]. Moreover, recent work presented in abstract form suggested that alternate splicing introduces new motifs affecting localization of Myo18Aα to dendritic spines of Purkinje neurons (http://researchfestival.nih.gov/2011/posters.cgi?id=CELLBIO-1).

Even though Myo18A belongs to the myosin family, it is not a typical mechano-enzyme as indicated by lack of ATP hydrolysis that normally couples myosin to the actin cytoskeleton [1, 7, 18]. Myo18Aα, however, appears to regulate cytoskeletal network interactions in subcellular membranes through binding different protein or lipid targets in different cell types [9, 19–22]. Studies in various mammalian cells have reported that Myo18Aαmodulates Golgi structure [21], budding of Golgi secretory vesicles [20, 21], and retrograde flow of cell membrane lamellipodia [22, 23]. In migrating cells, Myo18Aα localized to integrin adhesion complexes [19], and, in B lymphocytes, Myo18Aα localized with ezrin and the B cell receptor [9], suggesting roles for Myo18Aα in cell signaling processes. Interestingly, immune activation results in localization of SP-R210 on the surface of T lymphocytes [12]. On the other hand, the SP-R210_L_ and SP-R210_S_ cell-surface isoforms in macrophages assume a novel myosin function in recognition and uptake of SP-A opsonized bacteria [5, 8]. In addition to this opsonic function, studies in U937 cells, which exclusively express SP-R210_S_, indicated that SP-R210_S_ mediates endocytosis of SP-A [24].

SP-A has been shown to either bind or stimulate a number of receptors on macrophages [11, 25]. Different studies reported that SP-A could stimulate IgG Fc and complement-dependent phagocytosis of opsonized bacteria [26, 27]. Furthermore, SP-A was shown to also stimulate expression of non-opsonic receptors and phagocytosis through the macrophage mannose [28, 29] and scavenger receptors [30, 31]. Phagocytosis of SP-A-opsonized bacteria via SP-R210 is coupled to macrophage activation state as indicated by increased production of TNFα and nitric oxide [8, 13]; disruption of SP-R210_L_ abrogated phagocytosis of SP-A-opsonized bacteria [8] On the other hand, ligation of SP-R210 by free SP-A suppresses responses to inflammatory stimuli [12, 14, 24]. Binding of the SP-A collagen-like domain to the CD91/calreticulin receptor complex enhances uptake of SP-A-coated apoptotic cells and also results in pro-inflammatory responses [32]. SP-A, however, facilitates tonic suppression of alveolar macrophages under normal circumstances and helps restore resolution of inflammation by binding the immunosuppressive receptor SIRPα on alveolar macrophages [32, 33]. SIRPα suppresses downstream signaling through activation of SHP-1 phosphatase. Furthermore, binding of SP-A to SIRPα inhibits phagocytosis of apoptotic cells by alveolar macrophages through activation of SHP-1 and RhoA [33]. The globular carbohydrate recognition domain (CRD) of SP-A is responsible for binding to SIRPα [33]. The CRD domain of SP-R210 is also responsible for binding and suppressing pro-inflammatory CD14 and TLR pattern recognition receptors. In this regard, chronic exposure of human alveolar macrophages to SP-A and surfactant lipids increase expression of IRAK-M, which acts as an antagonist of TLR signaling [34]. Binding of SP-A to CD14 [35–37] and TLR-4 [38, 39] inhibits the inflammatory response to LPS, by a mechanism that alters trafficking of TLR-4 between golgi and endosomal vesicles in response to LPS [40]. On the other hand, earlier studies showed that SP-A enhances the ability of human macrophage cell lines to generate an inflammatory response as indicated by increased levels of the membrane receptors CD14, CD54, and CD11b, and elaboration of inflammatory cytokines [41–43]. In this case, surfactant lipids inhibited the inflammatory function of SP-A. Lack of SP-A alters the proteomic profile of alveolar macrophages in mice [44]. Importantly, pulmonary administration of human SP-A isolated from normal individuals [44] or alveolar proteinosis patients [45] to SP-A-deficient mice nearly restored the proteomic profile and rescued phagocytic activity of alveolar macrophages similar to WT mice, respectively, indicating that human and mouse SP-A modify alveolar macrophage phenotype and function similarly. In preceding studies, SP-A was shown to induce cross-tolerance or priming of differentiated monocytic cell lines as indicated by inhibition or enhanced secretion of different cytokines [46, 47], although the receptors were not identified. Interestingly, recent studies indicate that TLR-2 mediates several SP-A functions in suppressing responses to multiple inflammatory agents [48], in enhancing macrophage chemotaxis [49], and in modulating the timing of parturition [48, 50]. Taken together, SP-A utilizes diverse regulatory and counter-regulatory mechanisms to modulate innate immune functions of macrophages.

Here, we studied macrophages lacking expression of the SP-R210_L_ isoform. Previous findings revealed that dominant negative disruption of SP-R210_L_ blocked opsonic phagocytosis of SP-A-coated bacteria, but also enhanced non-opsonic phagocytosis via the scavenger receptor SR-A [8]. Our present findings indicate that SP-R210_L_ and SP-R210_S_ coordinate the function and expression of innate immune receptors in macrophages.

## Materials and Methods

### Reagents and antibodies

Chemicals were purchased from Sigma-Aldrich (St. Louis, MO). Pre-stained molecular weight markers were from Bio-Rad (Hercules, CA), and fetal bovine serum (FBS) from Atlanta Biologicals (Atlanta, GA). The TNFα ELISA kit was from eBioscience (San Diego, CA). Smooth lipopolysaccharide (LPS) from *Escherichia coli* serotypes O111:B6 or O26:B6 were from Sigma-Aldrich. The RNAeasy midi kit was from Qiagen (Valencia, CA). The High capacity cDNA reverse transcription kit and TaqMan qRT-PCR gene expression assays were from Life Technologies/Invitrogen (Carlsbad, CA). Fluorochrome conjugated monoclonal antibodies against CD11c (N418); CD11b (M1/70); CD14 (Sa2-8); CD282 (TLR-2; mT2.7); CD284 (TLR-4; UT41); SIRPα (P84); F4/80 (BM8); Ly-6C (HK1.4); TNFα(MPX-XT22), and CD16/32 Fc block (93) were from eBioscience (San Diego, CA). The CD36 (72–1) and CD284 (TLR-4; Sa15-21) antibodies were from BioLegend (San Diego, CA). The SR-AI/MSR1 (clone: 268318) and CD87 (uPAR; clone 109801) antibodies were from R&D (Minneapolis, MN). Isotype matched controls were from eBioscience or R&D. Unconjugated goat polyclonal against mouse CD14, TLR-2, CD36, SR-AI/MSR1, and rat monoclonal anti-mouse CD11b (M1/70) were purchased from R&D. Rabbit polyclonal antibodies against human SP-A were used as described previously [51]. Antibodies to SP-B were either a kind gift of Dr. Jeffrey Whitsett (Cincinnati Children’s Hospital Medical Center, Cincinnati, OH) or purchased through Seven Hills Bioreagents (Cincinnati, OH). Brefeldin A, and fix/permealizing solution for intracellular cytokine staining were from eBioscience. Antibodies to NFκB subunit RelA(p65), RelA(p65)^S536^, IRAK-1, and IκB were purchased from Cell Signaling. Secondary HRP-conjugated donkey anti-goat antibodies were from R&D. TrueBlot HRP-conjugated anti-rabbit and protein G-Sepharose IP beads were from eBioscience or GE Life Sciences. The ECL chemiluminescence kit was from Perkin Elmer (Waltham, MA). Monoclonal antibodies to SPR210 were generated by standard methods as previously described [52]. The characterization and purification of monoclonal SP-R210 antibodies will be reported elsewhere. Dynasore was obtained from SIGMA, and 5-(N-Ethyl-N-isopropyl) amiloride (EIPA) and NSC23766 were from SelleckChem through Fisher Scientific.

### Mice

WT C57BL/6 mice were purchased from Jackson Labs (Bar Harbor, MN). The SP-A^-/-^ mice were bred and maintained locally either at the University Of Cincinnati College Of Medicine or at Penn State College of Medicine. Mice were maintained in microisolator ventilated cages and provided autoclaved water and food ad libitum. SR-A^-/-^ transgenic mice at the two Institutions were derived independently as described previously and backcrossed [53–55] to the C57BL/6 genetic background. All procedures were in accordance to Institutional Animal Use and Care Committees.

### Isolation of Alveolar Macrophages

Alveolar macrophages were isolated by alveolar lavage using five consecutive washes of alveolar contents with 0.5 mL of PBS supplemented with 1 mM EDTA. Alveolar macrophages were collected by centrifugation and processed for Western blot analysis as described previously [56].

### Purification and characterization of human SP-A

SP-A was isolated from discarded therapeutic lung lavage from alveolar proteinosis patients by modifications [57] of the traditional butanol/octylglucoside extraction method of Haagsman and colleagues [58] (Method 1) or according to detailed protocols provided by Agrawal et al [48] (Method 2). SP-A preparations were dialyzed in 5 mM Hepes, pH 7.5, and stored frozen at -80°C until use. All procedures used LPS-free water from a Millipore water purification system (Millipore RiOs 16 and Milli-Q Biocell with resistance of >18.2 MΩ). The concentration of LPS in purified SP-A was measured using the Limulus Amebocyte Lysate QCL-1000 assay (Lonza, Walkersville, MA). LPS was undetectable in SP-A purified by Method 1. The concentration of LPS in SP-A prepared using Method 2 was 20 pg/μg of protein. Protein purity was determined by silver-staining as previously described [59]. For mass spectrometry, SDS-PAGE gels were stained with the Invitrogen SilverQuest staining kit. Proteins co-isolating with SP-A were excised, in-gel digested with trypsin and proteins identified by MALDI mass spectrometry at Penn State College of Medicine Mass Spectrometry Facility.

### Cell culture

The generation of control and SP-R210_L_(DN) Raw264.7 cells was recently described by us [8]. Briefly, cells were stably transfected with pTriex-2 vector expressing the carboxy-terminal domain of SP-R210 (SP-R210_L_(DN) cells [6]. Control cells were transfected with empty vector. Cells were cultured for 20–48 hrs in RPMI supplemented with 10% fetal bovine serum (FBS). Cells were cultured in 96-well dishes at a density of 50,000 cell/well or 12-well dishes at a density of 150,000–250,000 cells/well.

### Flow cytometry

Control and SP-R210_L_(DN) Raw264.7 cells were detached using non-enzymatic cell dissociation medium (SIGMA) and washed in PBS. After blocked in PBS, pH 7.4, supplemented with 1% goat serum, 0.5% BSA, and 5 μg/mL of Fc block at a concentration of 1x10^7^cells/ml for 1 hr on ice, cells were stained with recommended concentrations of monoclonal antibodies for 30 min on ice. For viability staining, cells were washed twice with PBS without protein or azide, and then stained with eBioscience e506 fixable viability dye for 20 min at 4°C. For intracellular cytokine staining, cells were washed once with FACS buffer (PBS containing 2% FBS, 0.02% sodium azide) and fixed with 100 μl eBioscience intracellular (IC) fixation buffer for 30 min at room temperature. Then cells were permeabilized with eBioscience permeabilization buffer, and stained with anti-mouse TNFα conjugated to phycoerythrin (PE). Events were acquired using either a BD FACS Calibur or LSR II flow cytometer (BD Pharmingen) and analyzed using FlowJo flow cytometry analysis software (Treestar, Mountain View, CA).

### Endocytosis assays

Control and SP-R210_L_(DN) cells were placed in 12 well plates at a density of 250,000 cells/well and cultured in RPMI/10% FBS for 20 hrs. The cells were then stimulated with 100 ng/mL or 2 μg/mL of LPS for CD14 and TLR-4 endocytosis assays, respectively, and harvested at 0, 1, 2, 3, and 4 hrs post-stimulation using cell dissociation buffer. Cells were blocked and then processed for cell surface staining with PE-Cy7-conjugated CD14 (eBioscience, clone Sa2-8) or PE-conjugated TLR4 antibody (BioLegend, clone Sa15-21) for 30 min at 4°C. To test the effect of inhibitors, cells were pretreated with 80 μM dynasore to inhibit dynamin, 40 μM EIPA to inhibit macropinocytosis, or 100 μM NSC23766 to inhibit RAC1 in normal medium 30 min prior to addition of LPS.

### Generation of cell extracts and Western blot analysis

Cultured cells were washed in PBS and detached using non-enzymatic cell dissociation medium. Cell suspensions were centrifuged at 210 x g at 4°C and lysed in ice-cold complete lysis buffer (50 mM Tris-HCl, pH 8.0, 150 mM NaCl, 1% NP-40 supplemented with 1 mM MnCl_2_, 10% glycerol, 1x Cell Signaling Phosphatase/Protease Inhibitor cocktail) by freeze and thaw cycles as described previously [12]. Extracts were used immediately, or stored frozen at -80°C. Proteins were separated on 4–17% SDS-PAGE gradient gels and transferred to nitrocellulose by semi-dry blotting. The blots were then blocked in Tris-buffered saline, pH 7.5, supplemented with 0.1% Tween 20, and 5% non-fat dry milk. Blots were probed with anti-Myo18A, CD14, SR-A, TLR-2, or CD36 antibodies and then incubated with of HRP-conjugated anti-rabbit or anti-goat secondary antibodies. Bound antibodies were visualized by enhanced chemiluminescence. Relative band intensity was determined by densitometry using a GS-800 Calibrated Densitometer (Bio-Rad) and Quantity One software (Bio-Rad).

### Confocal microscopy

Macrophages grown on glass coverslips were stimulated with 100 ng/mL LPS for set time points, washed with PBS, fixed for 15 min with 4% paraformaldehyde, permeabilized for 10 min in 0.3% Triton X-100/ PBS, and then blocked in 10% goat serum/ PBS for 60 min at room temperature. Subsequently, the cells were stained with rabbit p65(RelA) antibodies (1:400 dilution) and then with Cy3 conjugated anti-rabbit secondary antibodies (1:500 dilution). Coverslips were mounted on slides by using Prolong mounting medium with DAPI (Life Technologies). Confocal images of fluorescently labeled cells were acquired with a Leica AOBS SP8 laser scanning confocal microscope (Leica, Heidelberg, Germany) using a high resolution Leica 60X/1.3 Plan-Apochromat oil immersion objective at the Penn State College of Medicine Imaging Core. The laser lines used for excitation were continuous wave 405 (for DAPI) and 591 (for Cy3). These laser lines were produced by UV diode, 80 MHz white light laser (Leica AOBS SP8 module) respectively and the respective emission signals were collected sequentially using AOBS tunable filters. All images and spectral data measurement data were generated using the highly sensitive HyD detectors (with time gated option). The backscattered emission signals from the sample were delivered through the AOBS tunable filter (to remove irradiated laser), the detection pinhole set to 1 Airy unit (to obtain optimal lateral and axial resolutions), spectral dispersion prism, and finally to the HyD detectors. The width of the slits in front of each HyD could be software adjusted so that each HyD could detect spectral regions spanning from a 10-nm bandwidth up to the overall spectral capacity of the system (400–800 nm). Using this unique option, spectral scanning was performed on all the dyes to confirm signal specificity. Confocal images were analyzed using Imaris Software.

### Immunoprecipitation

Control and SP-R210_L_(DN) cells were cultured in 100 mm dishes for 24 hrs and then stimulated with 100 ng/mL LPS for 10, 30, 60 and 120 minutes. After set time periods, plates were washed twice with cold PBS. Cells were lysed directly on plate using complete lysis buffer solution on ice for 30 minutes and lysates were then harvested by centrifugation for 15 minutes at 10,000 x g at 4°C. Supernatants were collected without disturbing pellets. Protein concentration of supernatants was measured by BCA Assay kit (the kit information) and 1.5–2.0 mg of protein per sample was pre-incubated with pre-equilibrated Protein G Agarose beads (Roche) on a rotator for 3h at 4°C. Beads were removed by centrifugation at 12,000 x g for 1 minute and supernatants were transferred into fresh tubes. Pre-adsorbed lysates were then incubated with indicated antibodies or isotype controls on a rotator for 1h at 4°C and then 40–50 μL of pre-equilibrated Protein G Agarose beads (1:1, beads to bed volume) were added to lysates. Samples were incubated on rotator overnight at 4°C. Immunoprecipitation products were centrifuged for 1 minute at 12,000 x g and supernatant was discarded. Beads were washed three times in lysis buffer (50 mM Tris-HCl, pH 8.0, 150 mM NaCl, 1% NP-40). After the last wash, 2x Urea sample buffer (50 mM Tris-HCl, pH 6.8, 1.6% SDS, 7% glycerol, 8M Urea, 200 mM DTT, 0.01% bromophenol blue) was added directly onto beads. Prepared samples were incubated at room temperature for 20 minutes and heated at 95°C for 2 minutes. Samples were centrifuged at 12,000 x g for 1 minute, and then separated on 4–17% SDS-PAGE gels and analyzed by Western blotting.

### Quantitative real time RT-PCR

DNase-treated mRNA was isolated from control and SP-R210_L_(DN) macrophages using the Qiagen RNAeasy kit. cDNA was synthesized with the high capacity cDNA Reverse Transcription kit following the manufacturer’s protocol. Briefly, 1 μg of purified RNA was incubated with 2 μl of 10X buffer, 0.8 μl of 25X dNTPs, 2 μl of 10X random primers, 1 μl of RNAse inhibitor, and 50 U of reverse transcriptase in a final volume of 20 μl. The reaction was incubated for 10 min at 25°C, 2 hrs at 37°C, and inactivated for 5 min at 85°C. The cDNA was diluted five-fold prior to PCR amplification with TaqMan gene expression assays SR-A, CD11b, CD36, and CD14 and 18S ribosomal (r) RNA were quantified by real time RT-PCR (qRT-PCR) using TaqMan assays. SP-R210_L_ mRNA was measured using primers encompassing the PDZ domain containing exon 1 and exon 2 mRNA junction of the *Myo18A* gene. Common internal primers between exon 18 and 19 were used to quantify both SP-R210_L_ and SP-R210_S_ mRNA. Each 20 μl qPCR reaction included 10 μl of 2X TaqMan Gene Expression Master Mix, 1 μl of 20X TaqMan Gene Expression Assay, and a total of 10 to 40 ng of cDNA. The reactions were incubated in 384-well optical plates at 50°C for 2 min, 95°C for 10 min, and 40 cycles of 95°C for 15 seconds and 60°C for 1 min. Each sample was analyzed in triplicate along with no-template controls. Results were monitored and stored by the ABI PRISM 7900HT sequence detection system (Applied Biosystems) at the Functional Genomics Core Facility at the Penn State College of Medicine. Expression of mRNA for each gene was normalized to 18S rRNA. Data are expressed as relative mRNA expression in SP-R210_L_(DN) compared to control cells were calculated using the 2^-ΔΔCt^ method using the mean ΔCt in control cells as the calibrator [60].

### Statistics

Statistical comparison of data was performed using GraphPad Prism 5.0 software (San Diego, CA). Pair-wise comparisons using the Wilcoxon matched pairs t test were used to assess statistical differences. P<0.05 were considered significant.

## Results

### Dominant-negative inhibition of SP-R210_L_ mRNA in SP-R210_L_(DN) cells

**Fig 1:**
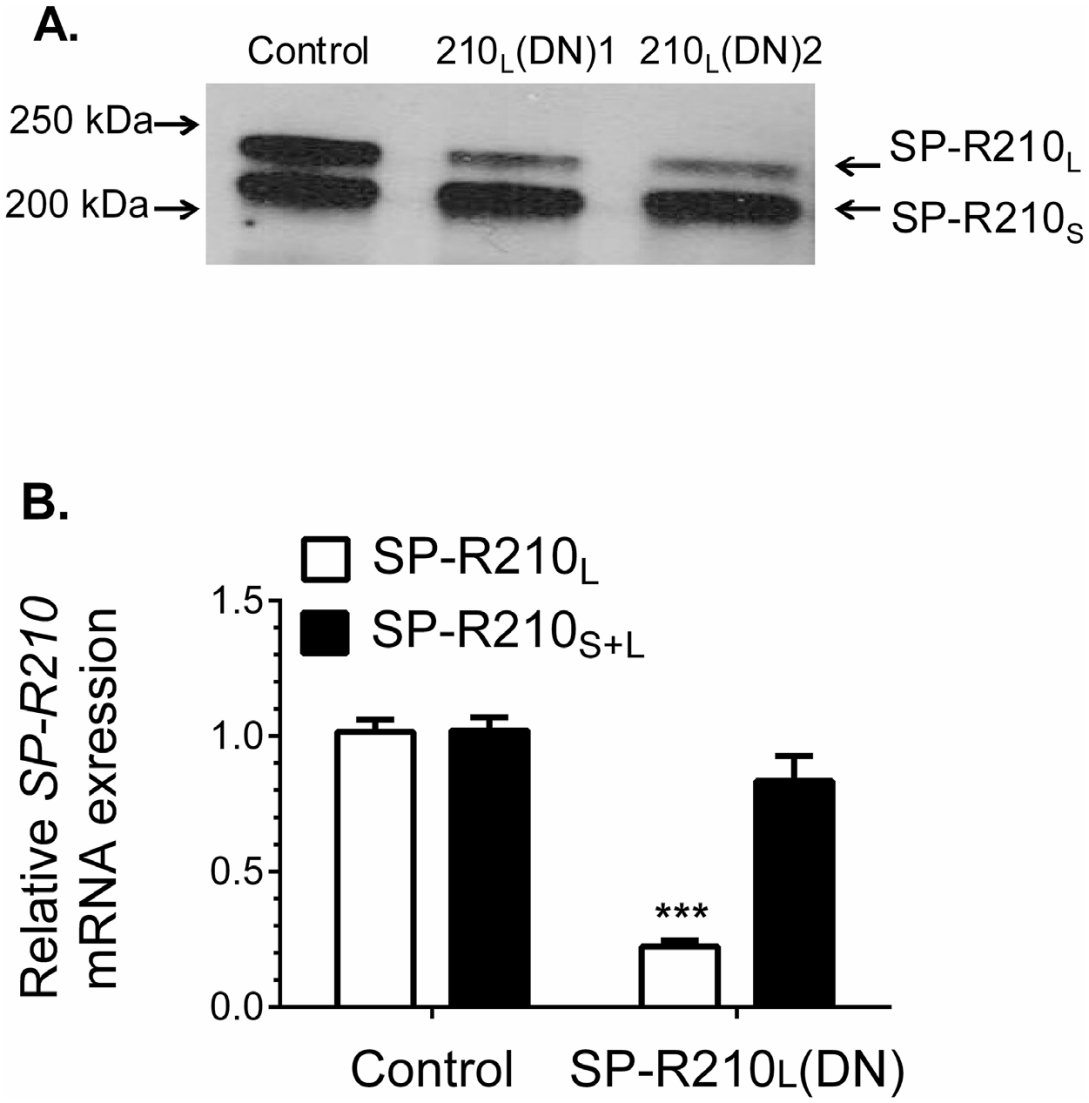
Dominant-negative disruption of SP-R210_L_. Raw264.7 cells were stably transfected with empty pTriexNeo2 control or vector containing the 300 (210_L_(DN1)) and 350 (210_L_(DN2)) bp cDNA of SP-R210 carboxy-terminal isoforms (6). A) Detergent extracts were analyzed by Western blotting using affinity purified polyclonal antibodies recognizing both SP-R210_L_ and SP-R210_S_. Lanes were loaded with 5 μg of protein. B) Total RNA from indicated cell lines was reverse transcribed and quantitated by qRT-PCR using TaqMan assays and primers encompassing the SP-R210_L_-specific exons 1 and 2 (red bars) and internal primers encompassing exon 17 and 18 common to both SP-R210 isoforms (black bars) and 18S rRNA as internal control. (n = 4 ***p<0.001).

Stable expression of the unique carboxy-terminal (ct) domain of SP-R210, SP-R210ct [5, 6], in Raw264.7 macrophages resulted in selective dominant-negative (DN) inhibition of SP-R210_L_ [8]. SP-R210ct isoforms differ by a 15 amino acid insertion [6] designated as SP-R210_L_(DN1) and SP-R210_L_(DN2) on Fig 1A. Western blotting (Fig 1A) and qPCR analysis (Fig 1B) demonstrate that stable expression of either SP-R210ct deletion mutant blocked both mRNA and protein expression of SP-R210_L_ by more than 85%. In contrast, expression of the SP-R210_S_ variant did not decrease significantly.

### Increased levels of innate immune receptors on the surface of SP-R210_L_(DN) cells

**Fig 2:**
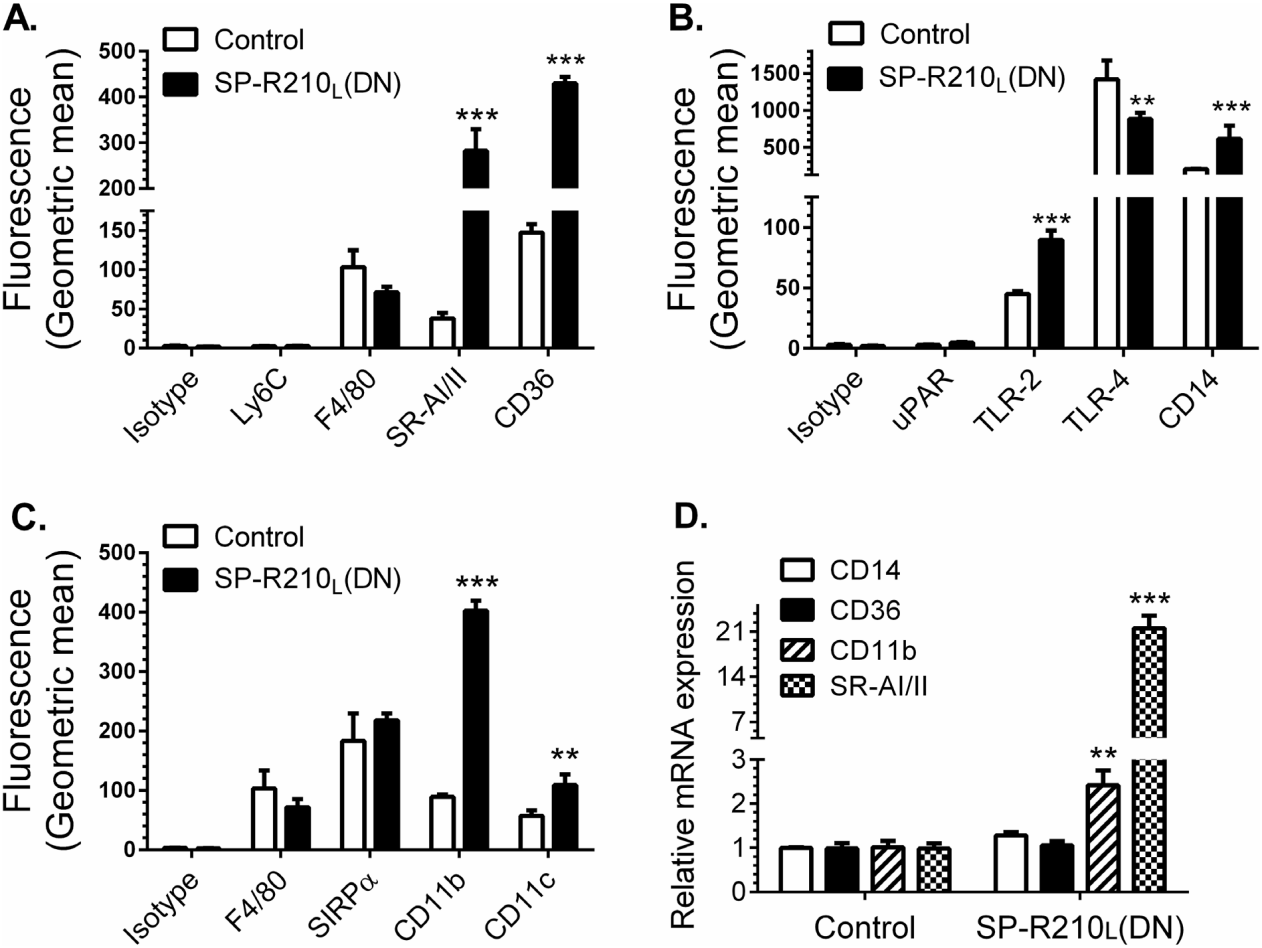
Depletion of SP-R210_L_ differentially enhances expression of innate immune receptors in macrophages. Control and SP-R210_L_(DN) cells were analyzed by flow cytometry using indicated APC (A) or PE-conjugated antibodies (B, C) (n = 4–8). (D) mRNA levels of indicated receptors in SP-R210_L_(DN) cells relative to control cells were determined by qRT-PCR (n = 4 independent experiments performed in duplicate, **p<0.02, ***p<0.005).

**Fig 3:**
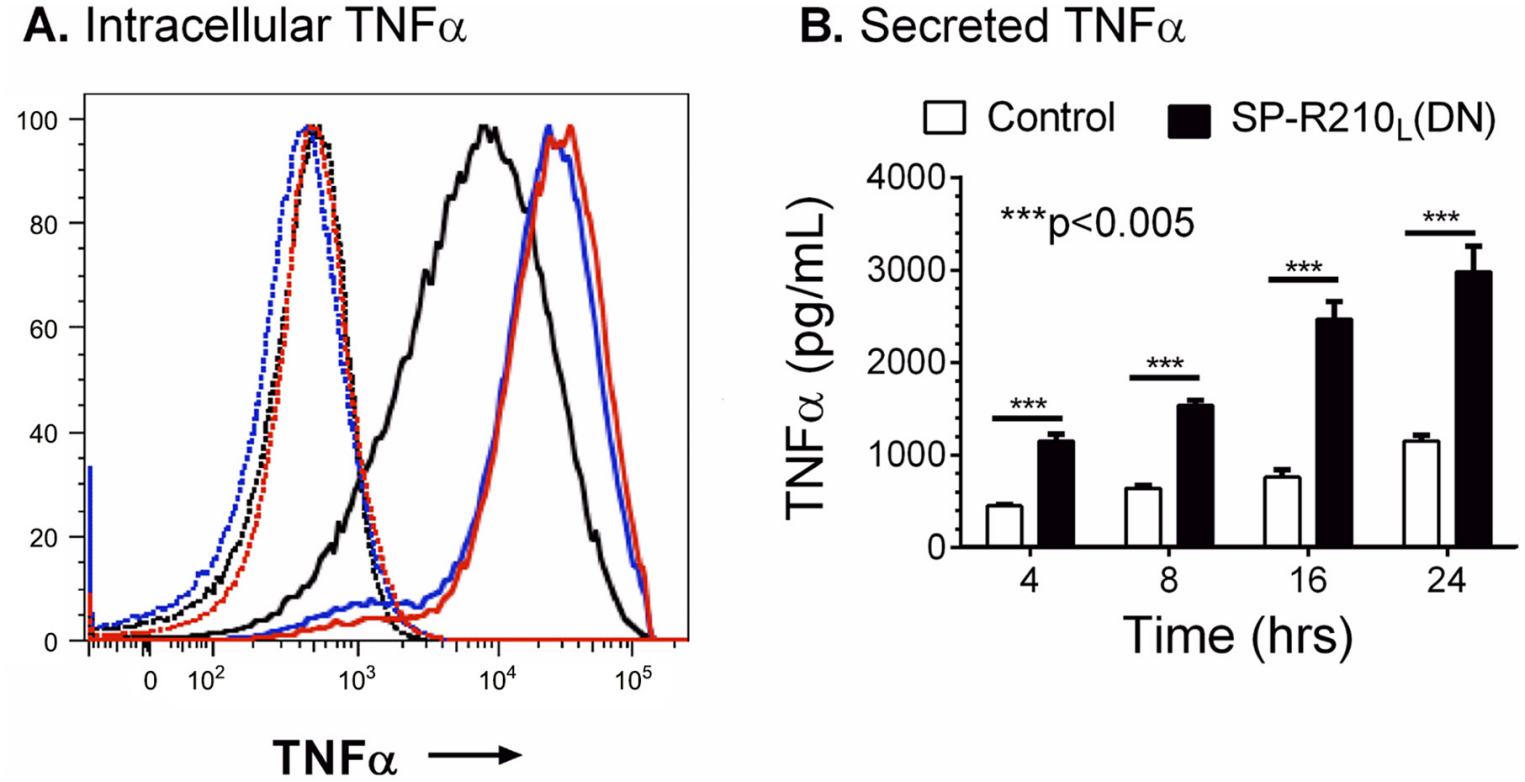
Increased responsiveness of SP-R210_L_(DN) cells to LPS. Control and SPR210_L_(DN) cells were plated in 12-well tissue culture dishes at a density of 150,000 cells/well and cultured 24 hrs in RPMI/10% FBS. Cells were then treated with 100 ng/mL LPS for 2, 4, 8, 16, and 24 hrs. A) Intracellular staining of TNFα was performed in brefeldin A blocked cells 2 hrs after treatment with LPS. Dotted histograms show untreated cells. Black, red, and blue histograms show intracellular TNFα staining of control, SP-R210_L_(DN)1, and SP-R210_L_(DN)2 cells. B) The levels of secreted TNFα were measured by ELISA in media at indicated time points after treatment with LPS. (n = 6; ***p<0.005).

We first asked whether disruption of SP-R210_L_ alters expression of innate immune receptors in addition to the previously described SR-A [8]. Studies below represent combined data from both SP-R210_L_(DN) cell lines. Fig 2 demonstrates 20- and 4-fold higher cell-surface levels of the scavenger receptor class A (SR-A) and CD36 (Fig 2A), 2- to 3-fold increase in TLR-2 and CD14 (Fig 2B), and 4- and 2-fold increases in CD11b and CD11c in SP-R210_L_(DN) cells (Fig 2C). Interestingly, the levels of TLR-4 were 40% lower than control in SP-R210_L_(DN) cells (Fig 2B). The monocytic marker Ly-6C and uPAR were expressed at low levels and were not different between control and SP-R210_L_(DN) cells (Fig 2A and 2B). The macrophage differentiation marker F4/80 was not statistically different compared to control cells (Fig 2A and 2C). Further, lack of SP-R210_L_ did not alter expression of SIRPα (Fig 2C). Interestingly, depletion of SP-R210_L_ resulted in 4- and 20-fold increases in mRNA levels of CD11b and SR-A (Fig 2D). The mRNA levels of CD14 and CD36 were similar to control cells (Fig 2D), even though surface expression of all four receptors increased significantly on SP-R210_L_(DN) cells. Given that CD14 levels increased in SP-R210_L_(DN) cells, we measured levels of TNFα after incubation with LPS. Control and SP-R210_L_(DN) cells were treated with 100 ng/mL smooth LPS to trigger macrophage activation via CD14. Intracellular staining 4 hrs after challenge with LPS demonstrates robust increase in the synthesis of TNFα in both SP-R210_L_(DN)1 and SP-R210_L_(DN)2 cells compared to controls (Fig 3A). Given that similar results were obtained with SP-R210_L_(DN)1 and SP-R210_L_(DN)2 cells, results described in SP-R210_L_(DN) are pooled data from both cell lines in subsequent studies. Fig 3B shows that SP-R210_L_(DN) cells secreted significantly more TNFα compared to control cells, consistent with higher levels of CD14. Accordingly, previous studies demonstrated increased functional activity of scavenger receptors [8], consistent with higher levels of SR-A and CD36 (Fig 2A) in SP-R210_L_(DN) cells. Taken together, these findings indicate that SP-R210_L_ acts as an intrinsic repressor of innate receptor expression and function through both transcriptional and post-transcriptional mechanisms.

### SP-A is a paracrine enhancer of SP-R210_L_ expression in alveolar macrophages

**Fig 4:**
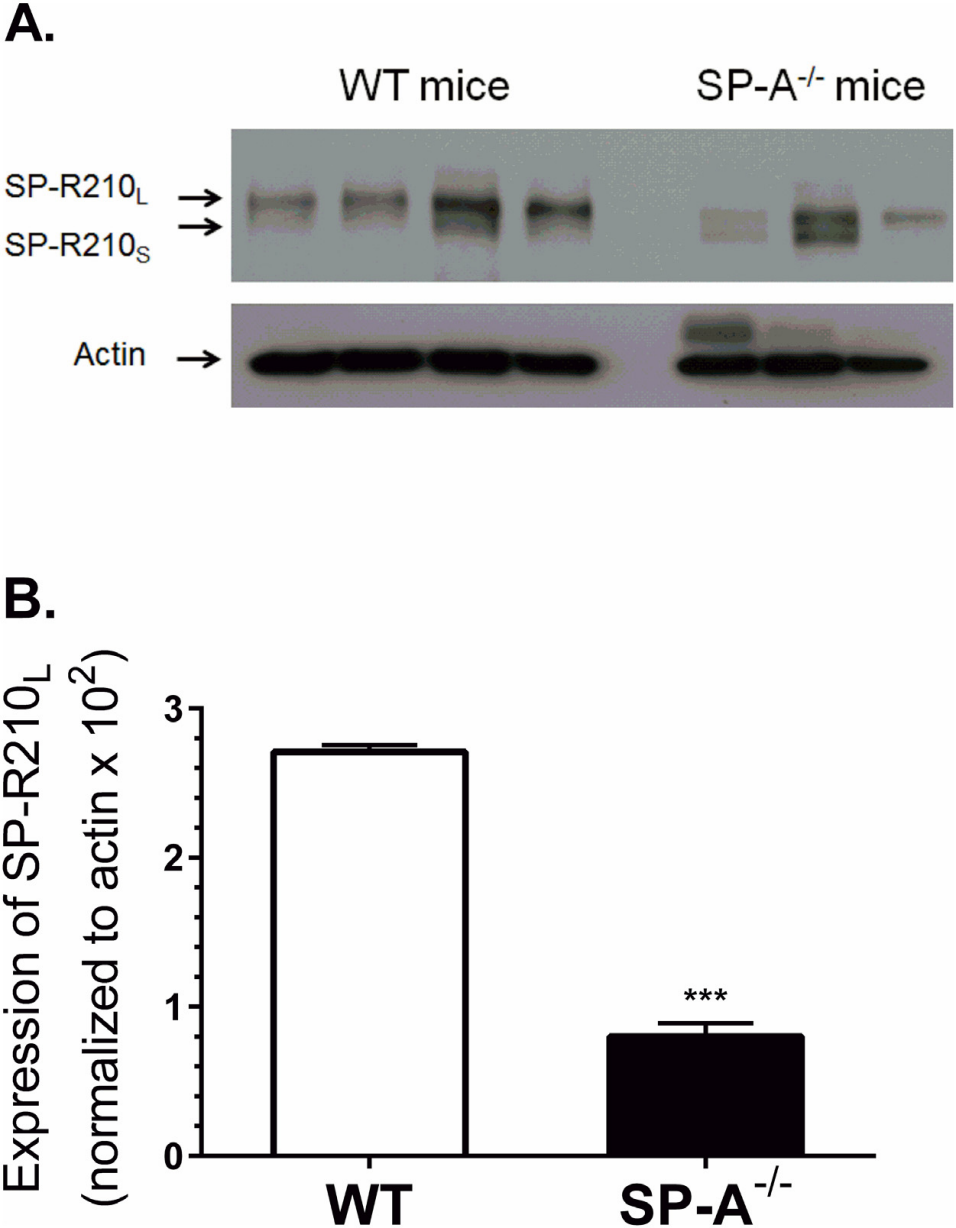
SP-A enhances expression of SP-R210_L_ in alveolar macrophages *in vivo*. Alveolar macrophages were isolated by lung lavage and processed for Western blot (A) and densitometry analysis (B) using polyclonal anti-SP-R210 antibodies. ***p<0.001, n = 8 WT mice and n = 6 SP-A-/- mice from two independent experiments.

To determine whether SP-A modulates SP-R210 expression *in vivo*, SP-R210 expression was assessed on alveolar macrophages from WT and SP-A^-/-^ mice. SP-R210_L_ is the main isoform on alveolar macrophages (Fig 4A) consistent with previous findings [8]. Notably, alveolar macrophages from most SP-A^-/-^ mice appear to express similar levels of both SP-R210_L_ and SP-R210_S_, suggesting differential expression of SP-R210 isoforms in the absence of SP-A *in vivo*. Densitometry analysis showed that WT macrophages express nearly five-fold higher levels of SP-R210_L_ compared to SP-A^-/-^ mice (Fig 4B). Similar results were obtained from the two original mouse lines maintained at the University of Cincinnati College of Medicine [61, 62] and Pennsylvania State University College of Medicine [55, 63, 64]. Consistently, treatment of Raw264.7 macrophages with SP-A *in vitro* also induced expression of SP-R210, although in this case both isoforms were induced (S1A Fig). SP-A did not induce expression of SP-R210_S_ in SP-R210_L_(DN) cells, suggesting that SP-R210_L_ is required for induction of SP-R210 by SP-A (S1B Fig). Together, these results indicate that SP-A is a paracrine regulator of SP-R210 expression in macrophages.

### SP-R210_S_ is a CD14 co-receptor

**Fig 5:**
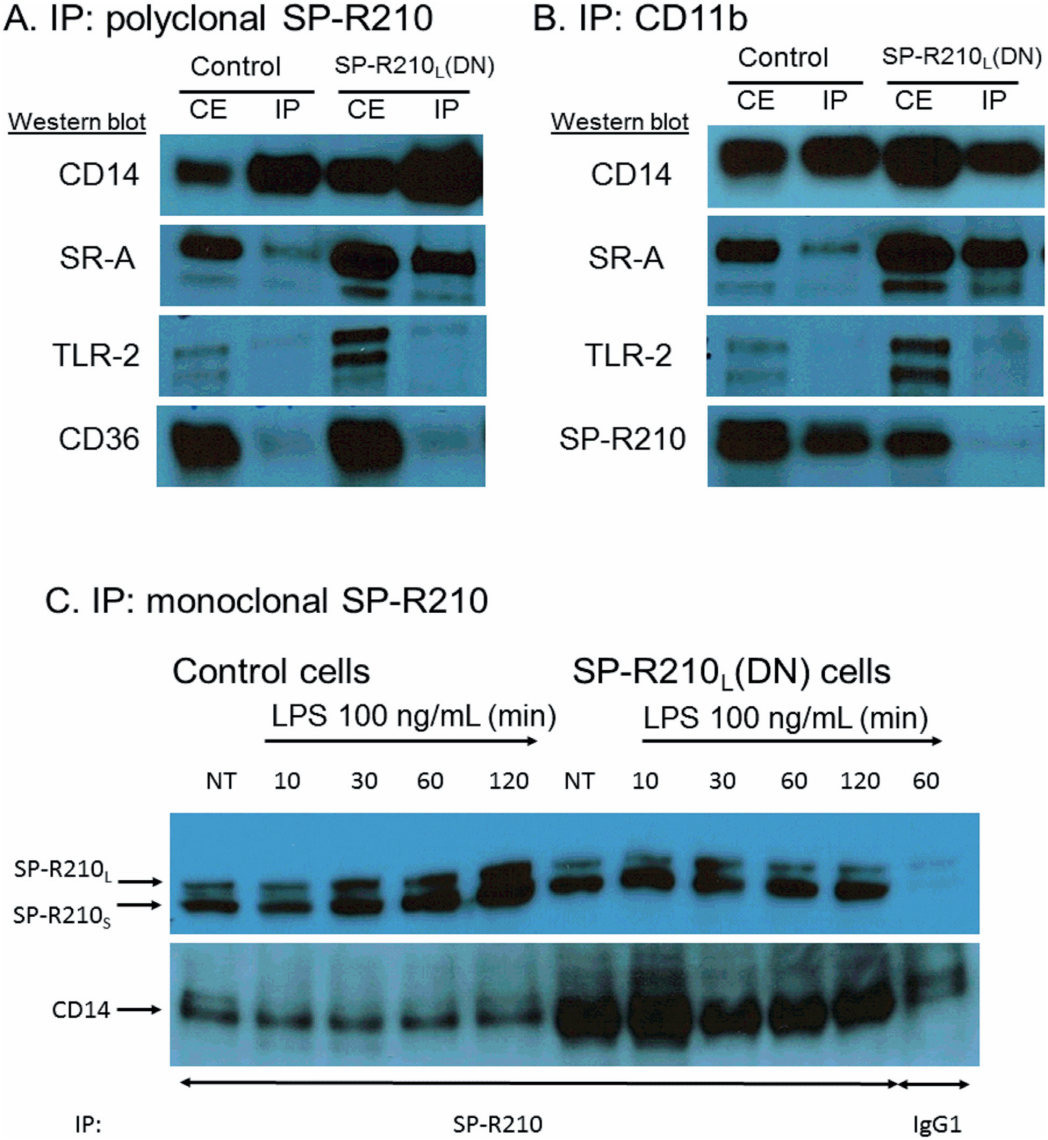
Interaction of SP-R210 with innate immune receptors. Immunoprecipitation experiments were carried out using 5 mg/mL (A and B) or 2.5 mg/mL (C) cell extracts from control and SP-R210_L_(DN) macrophages with polyclonal anti-SP-R210 (A) or monoclonal CD11b (B). To assess the effect of LPS treatment, immunoprecipitation reactions were carried out 10, 30, 60, and 120 min after treatment with 100 ng/mL LPS (C). Co-precipitated proteins were separated on SDS-PAGE gels and blotted with indicated antibodies. Extracts immunoprecipitated with monoclonal anti-SP-R210 were re-probed with polyclonal SP-R210 antibodies. A monoclonal IgG1 against an unrelated viral antigen served as control (C). Results shown are representative of 2–4 independent experiments.

To determine whether SP-R210 modulates LPS responsiveness via CD14, we performed immuno-precipitation experiments to assess whether SP-R210 interacts physically with CD14 and used neutralizing antibodies to assess responsiveness of control and SP-R210_L_(DN) macrophages to LPS. We also determined whether SP-R210 interacts with SR-A, CD11b, CD36, and TLR-2, proteins that are increased in SP-R210_L_(DN) cells (Fig 2 above). Of these, CD11b, TLR-2, and SR-A were also known to interact with either SP-A [27, 48, 49] and/or SP-R210 [8]. We used an affinity purified polyclonal anti-SP-R210 antibody recognizing both SP-R210_L_ and SP-R210_S_. Fig 5A shows that CD14 precipitated with SP-R210 antibodies in both control and SP-R210_L_(DN) cells. Co-precipitated CD14 was clearly enriched in SP-R210_L_(DN) cells. Interestingly, SR-A was also enriched in SP-R210_L_(DN) cells, suggesting that SP-R210_L_ controls the physical association between SP-R210_S_ and SR-A as well. In contrast, SP-R210 did not co-precipitate with CD36 and TLR-2 (Fig 5A) in neither control nor SP-R210_L_(DN) cells, which serves as an internal control for specificity of SP-R210 interaction with CD14 and SR-A. Reciprocal immuno-precipitation assays using monoclonal CD11b antibodies (Fig 5B) shows that CD11b interacts preferentially with SP-R210_S_ in control cells. Interestingly, CD11b and SP-R210_S_ did not co-precipitate in SP-R210_L_(DN) cells. As a member of the myosin family, SP-R210 is expected to form dimers via the carboxy-terminal coiled-coil domain. However, the preferential, albeit partial, interaction of CD11b with the short SP-R210_S_ but not the longer SP-R210_L_ isoform suggests that SP-R210_L_ and SP-R210_S_ do not form heterodimers with one another in macrophages [1]. Similar to SP-R210 (Fig 5A), CD11b did not associate with TLR-2 (Fig 5B) or with CD36 (not shown). Immunoprecipitation experiments using monoclonal antibodies to SP-R210 show that SP-R210s and CD14 form a stable complex before and after treatment of cells with LPS over two hrs (Fig 5C). Interestingly, LPS treatment increased the level of immunoprecipitated SP-R210 isoforms over time. The level of co-precipitated CD14 in control cells was lower than in SP-R210_L_(DN) cells, suggesting that SP-R210_L_ controls association of SP-R210_S_ with CD14 (Fig 5C).

**Fig 6:**
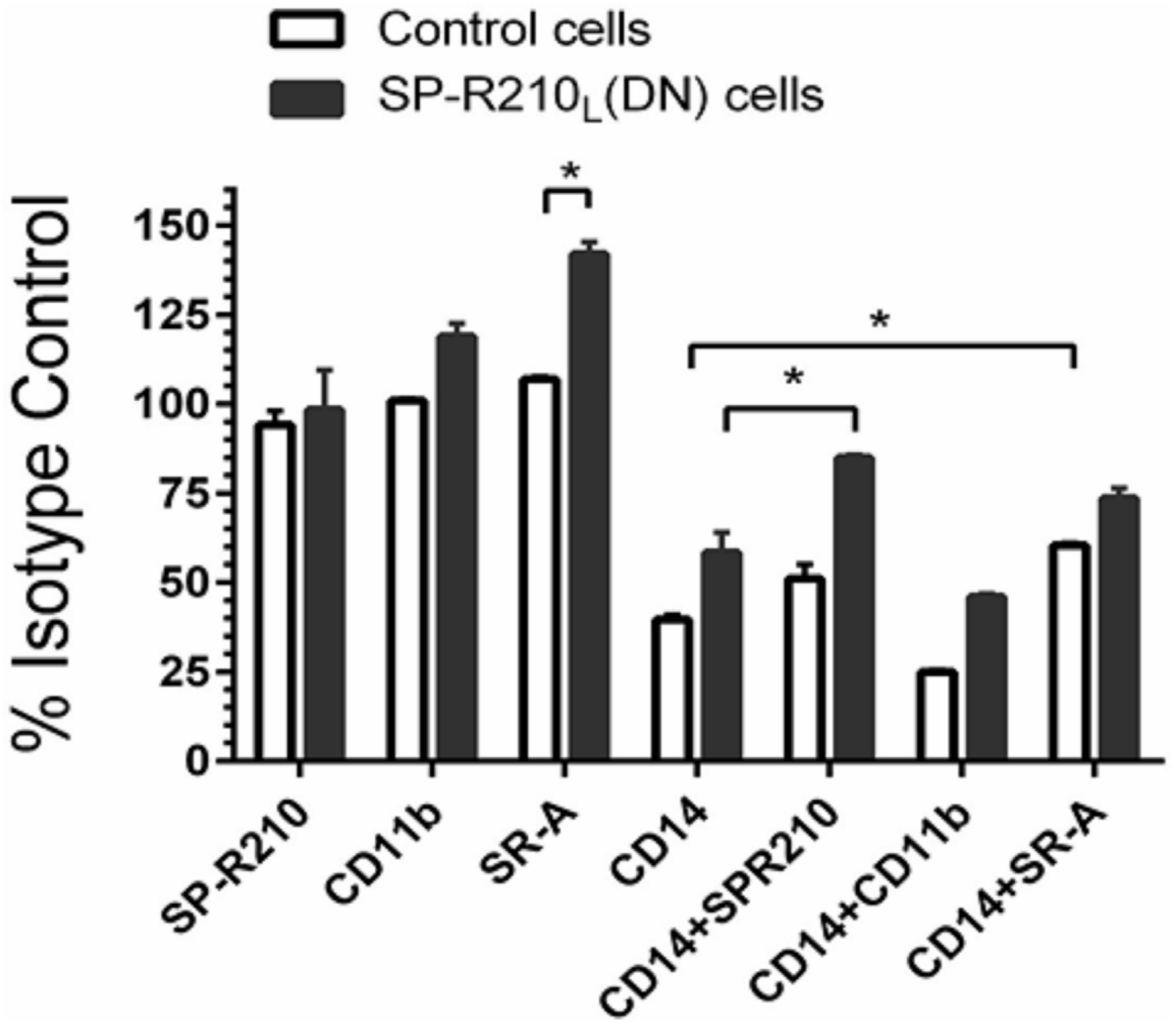
Effect of neutralizing antibodies on the inflammatory response to LPS. Control and SP-R210_L_(DN) macrophages were pretreated for 60 min with 20 μg/mL of indicated individual or antibody combinations or respective isotype controls followed by stimulation with 100 ng/mL of LPS for 4 hrs. Cells were then harvested and processed for intracellular cytokine staining with TNFα antibodies. Stained cells were analyzed by flow cytometry. The mean fluorescence of positively stained cells was expressed as percent of isotype control treated cells. Data shown are means±SD and are pooled from 2–4 independent experiments performed in triplicate. *p<0.05.

To evaluate the functional significance of these interactions, we used antibodies to assess activation of macrophages by LPS (Fig 6). Cells were pre-treated with antibodies and then with 100 ng/mL LPS for 4 hrs followed by intracellular staining for TNFα. Pretreatment of macrophages with SP-R210 and CD11b antibodies did not affect TNFα synthesis in LPS-stimulated control cells. However, SR-A antibodies stimulated TNFα significantly in SP-R210_L_(DN) compared to control cells by about 30% (Fig 6). CD14 antibodies blocked TNFα by 50% in both cell lines. Combined treatment with SP-R210 or SR-A antibodies interfered with the ability of CD14 antibodies to inhibit TNFα synthesis in SP-R210_L_(DN) cells consistent with a close physical proximity between SP-R210_S_, CD14 and SR-A in SP-R210_L_(DN) cells as predicted by the immunoprecipitation results above. Consistent with a lack of interaction of SP-R210 with CD11b (Fig 5B), the CD11b antibody did not interfere with the CD14 inhibitory effect (Fig 6). Taken together these results indicate that SP-R210_L_ intrinsically regulates formation of pro-inflammatory innate immune receptor complexes between SP-R210_S_, SR-A, and CD14.

### SP-R210 isoforms regulate NFκB activation downstream of TLR-4

**Fig 7:**
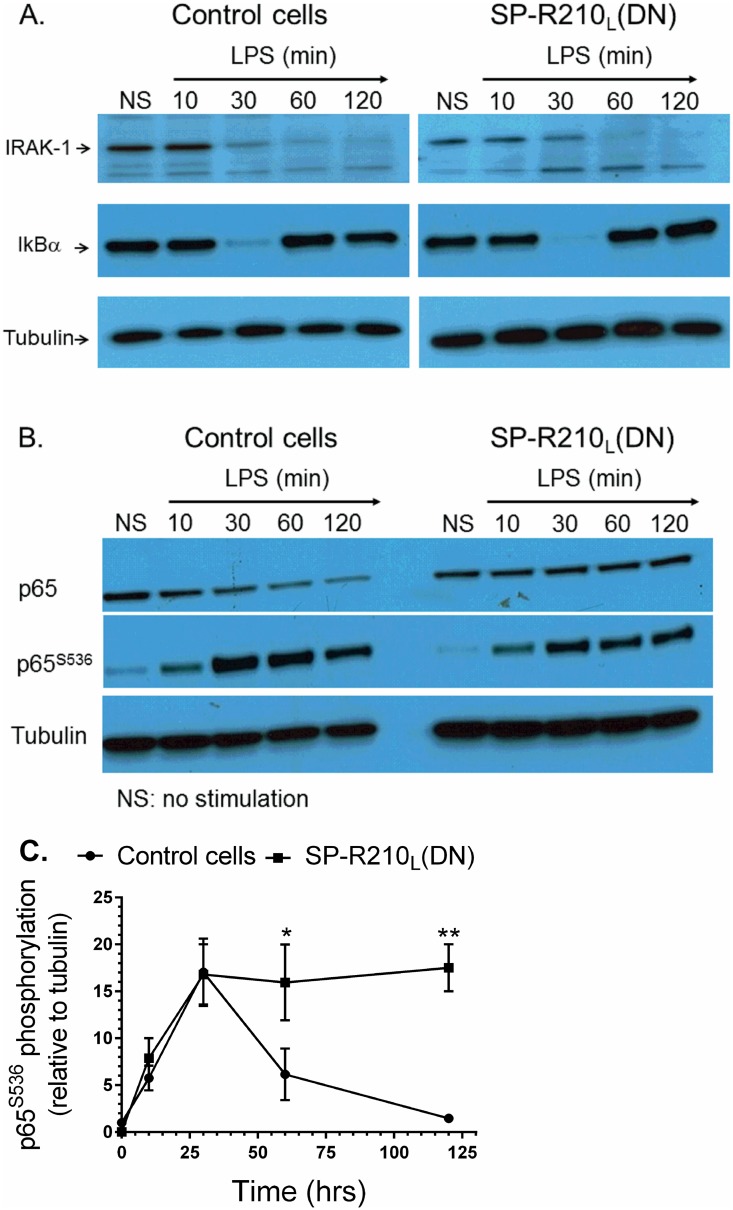
Activation of TLR4 signaling in control and SP-R210_L_(DN) cells. Macrophages were stimulated with 100 ng/mL LPS and harvested at indicated time points. Non-stimulated (NS) and stimulated cells were harvested and processed for Western blot analysis. Blots were probed with IRAK-1 (A) or NFκB p65 (B) antibodies, stripped, and then reprobed with IκB or phosphorylated p65 antibodies, respectively. Blots were stripped and then re-probed with tubulin as loading control. Densitometry analysis compared the levels of phosphorylated p65 relative to tubulin (C). Blots in A and B are representative of 2–4 independent experiments. Data shown in C are means±SD, n = 2- independent experiments. *p<0.05.

**Fig 8:**
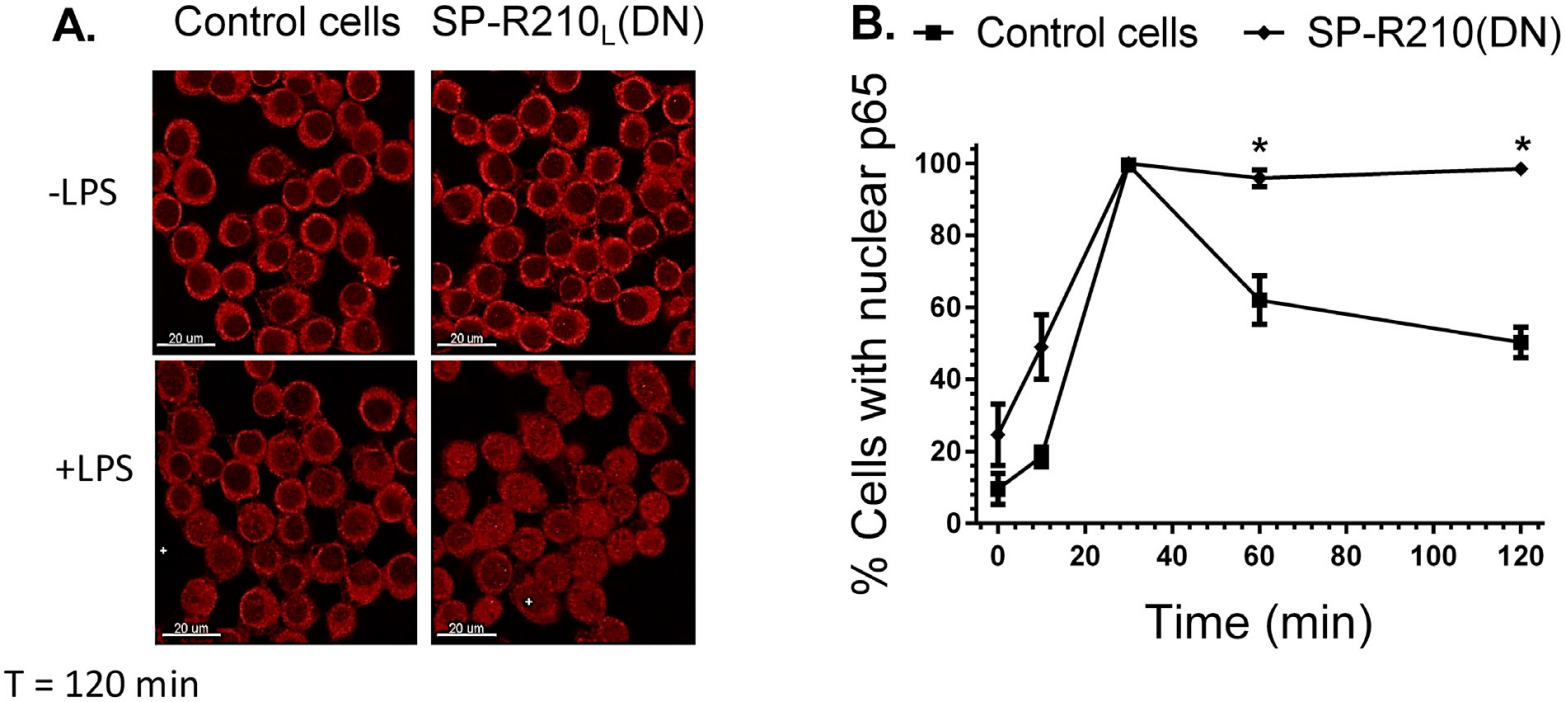
Nuclear translocation of NFκB in control and SP-R210_L_(DN) cells. Macrophages were grown on glass coverslips for 24 hrs, and then stimulated with 100 ng/mL LPS. Stimulated cells were then processed for immunofluorescent staining with anti-p65 NFκB at indicated time points after LPS treatment. The nuclear localization of NFκB was visualized by confocal microscopy (A). The percentage of cells containing nuclear p65 was quantitated in 10 random microscopic fields at 100 x magnification (B). Data shown are means±SD, n = 2 independent experiments performed in duplicate. *p<0.05.

Previous studies have shown that LPS binds CD14 and transfers LPS to the toll-like receptor TLR-4 resulting in nuclear translocation and activation of the transcription factor NFκB [65]. The proximal TLR-4 signaling pathway involves myddosome formation followed by activation and degradation of IRAK-1 and downstream phosphorylation and degradation of IκB [66]. Degradation of IκB allows phosphorylation and translocation of NFκB p65 (RelA) subunit to the nucleus. Restoration of IκB expression contributes to termination of NFκB signaling [67]. Therefore, we determined whether lack of SP-R210_L_ alters TLR-4 signaling. The Western analysis on Fig 7A demonstrates that the kinetics of IRAK1 and IκB degradation were similar between control and SP-R210_L_(DN) cells. Expression of IκB was restored after 30 min of LPS stimulation in both cell lines. However, the Western and densitometry analyses of Fig 7B and 7C demonstrated that phosphorylation of NFκB at serine 536 was transient in control cells but remained elevated in SP-R210_L_(DN) cells. Furthermore, confocal fluorescent microscopy analysis on Fig 8A and 8B shows prolonged retention of NFκB in the nucleus of SP-R210_L_(DN) cells compared to controls. These results indicate that SP-R210 regulates duration of NFκB signaling without affecting early signaling events of TLR-4 activation.

### SP-R210_L_ and SP-R210_S_ mediate distinct internalization mechanisms of CD14

**Fig 9:**
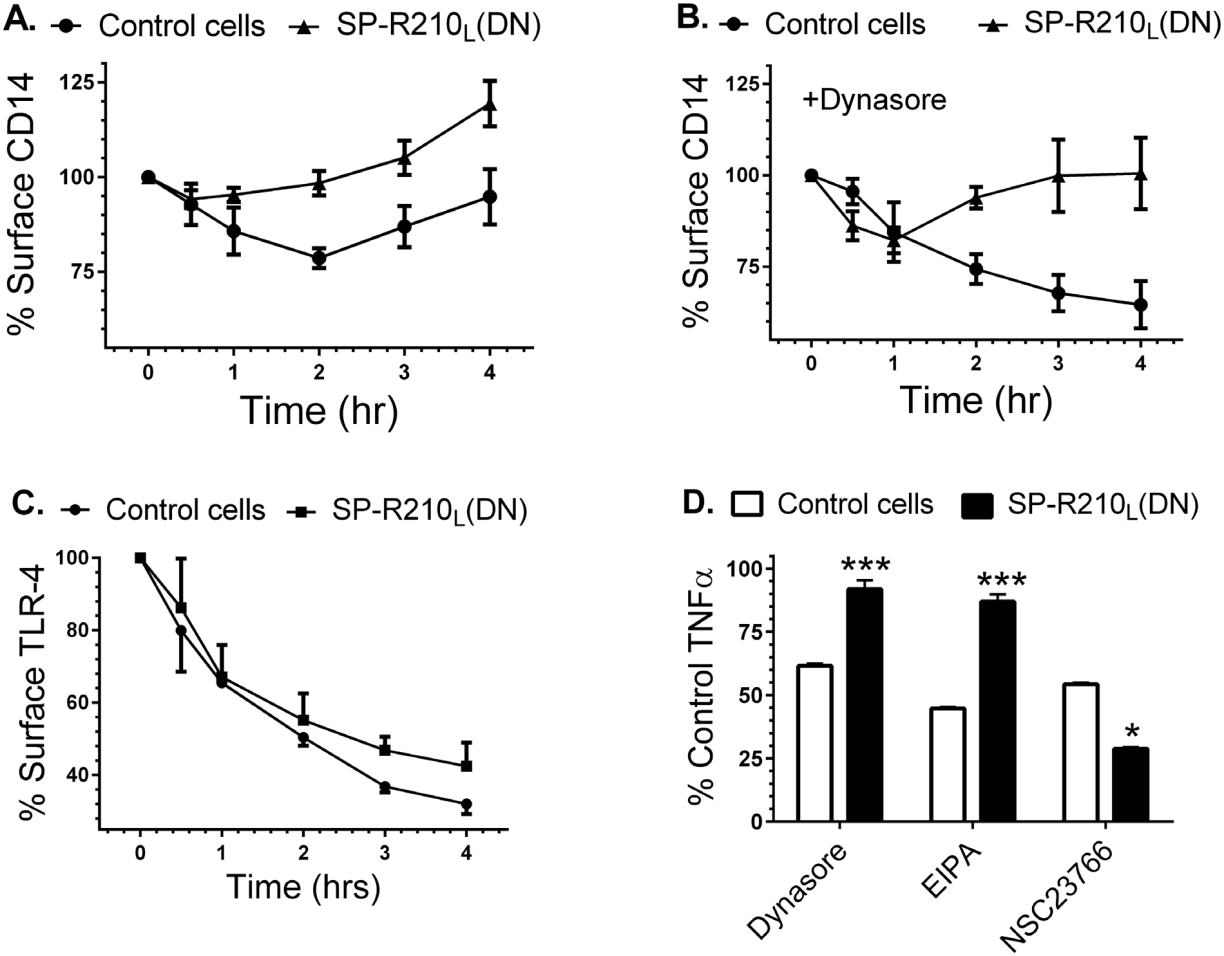
Effect of SP-R210_L_ disruption on CD14 and TLR-4 internalization and signaling. Macrophages cultured for 24 hrs on 12 well plates were simulated with 100 ng/mL LPS for indicated time points. The cells were then harvested at indicated time points using non-enzymatic cell displacement medium and stained with antibodies to CD14 (A and B) or TLR-4 (C). The effect of dynasore on internalization of CD14 (B) and of dynasore, EIPA, and NSC23766 on TNFα synthesis (D) was assessed by addition of inhibitors 30 min before addition of LPS. Harvested cells were analyzed by flow cytometry and mean fluorescence was expressed as % of control compared to non-stimulated control or SP-R210_L_(DN) cells (t = 0) (A-C) or as percent of control TNFα in SP-R210_L_(DN) cells compared to control cells (D). Data shown are means±SD, n = 2–4 independent experiments performed in triplicate. ***p<0.01, *p<0.04.

It has been demonstrated that CD14 controls internalization of TLR-4 which may determine TLR-4 activation from either the cell surface or endocytic vesicles [66]. CD14 was also shown to mediate macropinocytosis-mediated clearance of LPS [68]. We thus asked whether SP-R210 variants influence trafficking of CD14. Control and SP-R210_L_(DN) macrophages were treated with 100 ng/mL LPS overtime to monitor internalization of CD14. Fig 9A demonstrates that 25% of CD14 was lost from the cell-surface by 2 hrs after addition of LPS and then new or recycled CD14 returned to the cell-surface by 4 hrs. In contrast, CD14 was replenished faster in SP-R210_L_(DN) cells (Fig 9A); only 15% of CD14 was lost from the cell-surface by 30 min with surface CD14 quickly returned to the cell-surface above the levels of unstimulated SP-R210_L_(DN) cells. To probe the trafficking of CD14 further, we monitored surface CD14 after addition of dynasore, an inhibitor of clathrin and dynamin-dependent endocytosis [69]. Dynasore can also inhibit fluid phase endocytosis [70], endosomal recycling [71], and constitutive protein secretion in macrophages [70–72]. Fig 9B demonstrates that dynasore did not block internalization of CD14 in control cells, consistent with dynasore-insensitive macropinocytosis of CD14. However, dynasore blocked replenishment of surface CD14 completely in control cells and partially in SP-R210_L_(DN) cells, suggesting that dynasore inhibits secretion of newly synthesized CD14. Dynasore, however, reveals a distinct trafficking process in SP-R210_L_(DN) cells that is characterized by internalization over the first hour after addition of LPS followed by return of CD14 to the cell surface (Fig 9B), although additional studies will be needed to distinguish whether this represents dynasore-insensitive recycling or secretion of CD14. Differences in trafficking of CD14, however, do not impair endocytosis of TLR-4, although internalization of TLR-4 in SP-R210_L_(DN) cells slowed after 1 hr of LPS compared to controls (Fig 9C). Dynasore has been shown to block endocytosis of TLR-4 inhibiting signaling from endocytic compartments [73]. CD14, however, was shown to mediate endocytosis of LPS via macropinocytosis [68]. Therefore, we compared the effects of dynasore and the macropinocytosis inhibitor EIPA [74] on the inflammatory response to LPS using intracellular TNFα as a readout of the LPS response. Fig 9D shows that dynasore and EIPA inhibited TNFα by 40 and 60%, respectively, indicating that internalization of CD14 is required to mediate part of the inflammatory response in control cells. In contrast, SP-R210_L_(DN) cells were insensitive to inhibition by both dynasore and EIPA (Fig 9D). Previous studies have shown that the small GTPase Rac1 mediates macropinocytosis in macrophages [75, 76]. Accordingly, NSC23766 an inhibitor of the small GTPases Rac1 and Rac2 blocked TNFα production in both control and SP-R210_L_(DN) cells. Interestingly, NSC23766 was significantly more effective at inhibiting TNFα in SP-R210_L_(DN) compared to control cells (Fig 9D). Taken together, these results indicate that SP-R210_L_ and SP-R210_S_ isoforms mediate trafficking of CD14 through distinct macropinocytosis-like mechanisms.

### SP-A preparation influences responsiveness of macrophages to LPS

**Fig 10:**
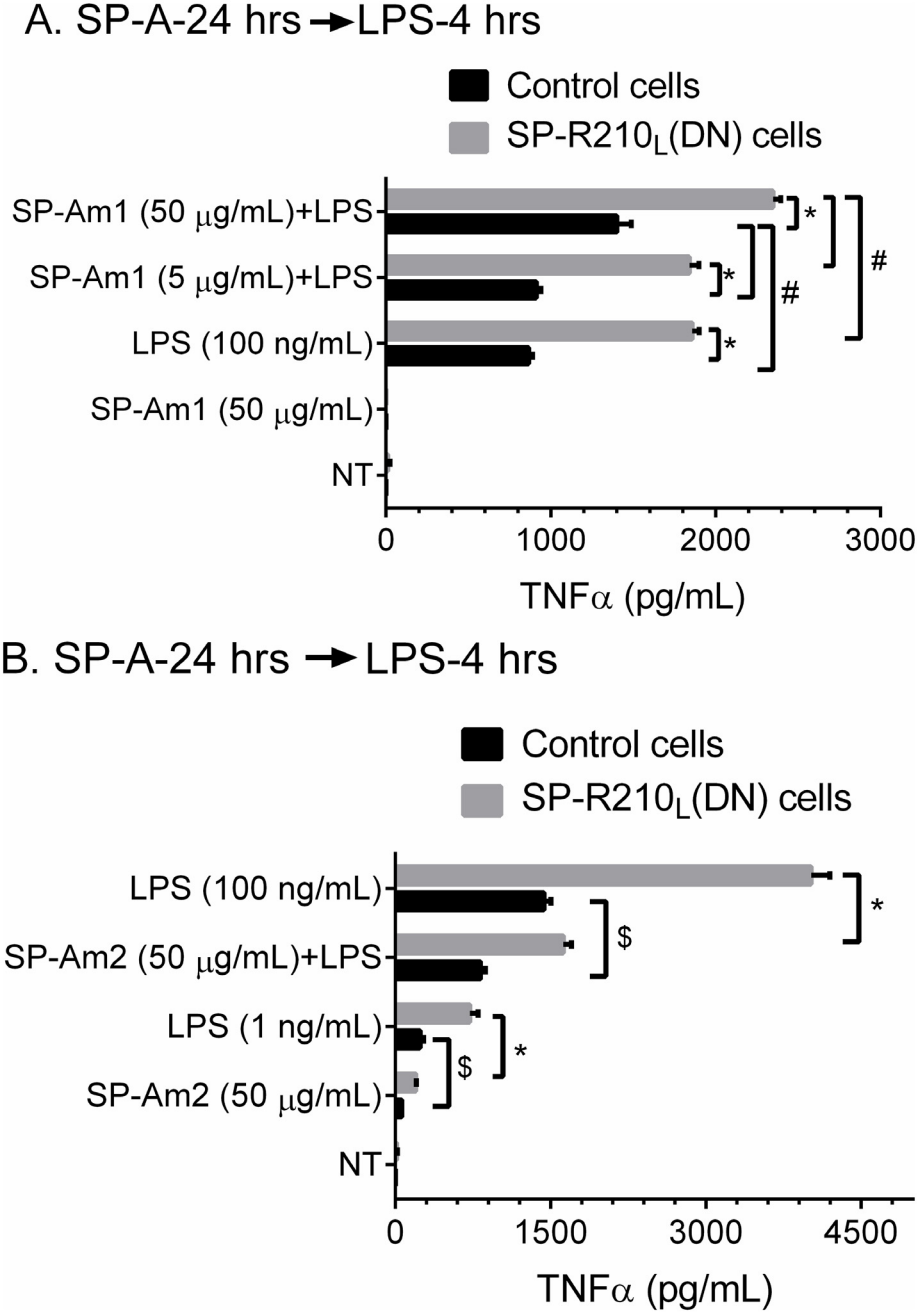
SP-A and SP-R210_L_ modulate responsiveness of macrophages to LPS. Control and SP-R210_L_(DN) cells were pretreated with SP-A purified by either method 1 (SP-Am1) or method 2 (SP-Am2) as described in “Materials and Methods”. Cells were pretreated with indicated amounts of SP-Am1 (A) or SP-Am2 (B) for 24 hrs and then incubated with 100 ng/mL LPS. Levels of secreted TNFα were measured in media by ELISA at 4 hrs after addition of LPS. Data shown are means±SD, n = 3 representative of 3–6 independent experiments. Lines indicate significant differences between indicated groups at *p <0.0001; ^#^p<0.04; ^$^p<0.005

We then assessed whether SP-A modifies the inflammatory response to LPS. For these studies, we used SP-A from alveolar proteinosis fluid from the same individual purified by either a modified [57] butanol/octylglucoside method (SP-Am1) [58] or the isopropyl ether/butanol extraction method (SP-Am2) as recently described [48]; SP-Am1 as purified here has been shown to enhance macrophage activation [57, 77] whereas SP-Am2 was shown to be a potent antagonist of multiple toll-like receptors in several macrophage lines including Raw264.7 macrophages [48]. Control and SP-R210_L_(DN) macrophages were exposed to low, 5 μg/mL, or high, 50 μg/mL, SP-Am1 for 24 hrs and subsequently incubated with 100 ng/mL of LPS (Fig 10A). Fig 10A shows that SP-Am1 enhanced responsiveness to LPS in both control and SP-R210_L_(DN) cells. SP-Am1 alone had no effect. However, SP-Am1 alone induced expression of CD14 in control cells but did not alter expression of CD14 in SP-R210_L_(DN) cells (S2 Fig). The effect of SP-Am1 on LPS responsiveness appears long term since it was observed 24 hrs after LPS treatment (S3A Fig). In contrast to SP-Am1, Fig 10B shows that SP-Am2 inhibited LPS-induced TNFα of both cell lines. The effect of SP-Am2 was examined at 50 μg/mL concentration (Fig 10B). SP-Am2 contained measurable levels of LPS, which translates to 1 ng/mL LPS at the 50 μg/mL SP-A dose. Compared to SP-Am1 alone that did not induce TNFα (Fig 10A), treatment with SP-Am2 produced measurable TNFα in both control and SP-R210_L_(DN) although at 20–30% lower level of an equivalent amount of LPS alone (Fig 10B).

To address the different response to LPS in SP-Am1 and SP-Am2-treated macrophages, we evaluated the purity of SP-A preparations from the same and different individuals by silver staining, Western blot analysis, and mass spectrometry (S4A–S4C Fig). Silver-staining using the formaldehyde/glutaraldehyde based protocol of Rabiloud et al [59] revealed that SP-Am2 co-isolated with higher levels of low molecular weight proteins of approximately 8–15 kDa (S4A Fig) in the molecular range of surfactant protein B (SP-B). Of note, SP-B is not detectable by Coomassie blue staining [78] and thus SP-A preparations stained with Coomassie would appear pure. We obtained similar results with SP-A from three different individuals. Western blot analysis confirmed the presence of SP-B co-isolating with both SP-Am1 amd SP-Am2, although clearly prominent in the SP-Am2 preparation (S4B Fig). Silver staining with the mass spectrometry compatible SilverQuest from Invitrogen revealed additional protein species above 300 kDa and proteins in the 8–25 kDa range (S4C Fig). The low molecular weight proteins are prominent in the SP-Am2 preparation with one protein in bands 5 and 6 that is present in one SP-Am1 and both SP-Am2 preparations (S4C Fig), respectively. These protein bands in SP-Am1 and SP-Am2 from APF-1 proteinosis material were in-gel digested and identified by MALDI mass spectrometry. The high molecular weight protein in bands 1 and 2 were similar in the two preparations and were both identified as gp340, a known binding protein for SP-A [79]. Band 3 contains a fragment of SP-A and ferritin light chain (S4C Fig), a previously described contaminant in SP-A preparations [5]. Ferritin light chain has been shown to suppress LPS-induced activation of macrophage [80]. Ferritin may contribute to the early inhibitory effect after brief 1 hr pre-treatment of macrophages with SP-Am1 and 4 hr stimulation with LPS (S3B Fig), although SP-Am1 retained its priming effect compared to SP-Am2 as observed 24 hrs after treatment with LPS (S3B Fig). Bands 4 in SP-Am1 and bands 6, 7, and 8 in SP-Am2 all contain SP-B. Band 5 contains the lung-specific aspartyl protease napsin A. In addition to SP-B, bands 6 and 7 also contain napsin A and the nuclear Histone H4, respectively. A blank piece of gel (band 10) did not yield any protein identifications. Intracellular napsin A has been shown to process pro-SPB in lamellar bodies of alveolar type II epithelial cells [81], although secreted napsin A may degrade cell surface proteins on alveolar cells [82]. The 30 kDa band 9 only contained SP-A as expected. All proteins were identified at 100% confidence index. These studies indicate that SP-A treatment leading to either enhanced or reduced responsiveness *in vitro* can depend in part on the level of co-isolating bioactive surfactant components or other molecules in different SP-A preparations.

## Discussion

Precise regulation of the innate immune system is of paramount importance to respiratory health. Expression and sub-cellular localization of innate immune receptors determines the outcome of signaling responses that coordinate inflammation with clearance of pathogens. The present findings demonstrate that the SP-A receptor SP-R210_L_ isoform is an intrinsic modulator of innate immune receptors in macrophages. Multiple macrophage receptors have been shown to interact with SP-A [11, 25, 83], although the mechanisms by which SP-A modulates macrophage functions remain obscure. We found that SP-R210_L_ disruption leads to 2-20-fold increased expression of several innate immune receptors at both protein (TLR-2, CD11c, CD36, and CD14) and transcriptional (SR-A, CD11b) levels. Studies on signaling pathways indicate that SP-R210_S_ and SP-R210_L_ control activation and deactivation of the transcription factor NFκB, respectively. Importantly, studies in alveolar macrophages from SP-A^-/-^ mice indicate that SP-A works in a paracrine fashion to maintain optimal expression levels of SP-R210_L_ [8], thereby modulating the functional phenotype of alveolar macrophages *in vivo*.

Our results indicate that SP-R210 isoforms regulate macrophage activation by CD14. Previous studies demonstrated that LPS binding to CD14 induces macropinocytosis and degradation of LPS in macrophages [68, 84, 85]. In turn, CD14 is required for LPS-induced endocytosis and activation of TLR-4 signaling in endosomes [66, 73]. Here, disruption of SP-R210_L_ revealed distinct mechanisms of CD14 uptake that modulate threshold and duration of macrophage activation in response to LPS. In addition to TLR-4 [65, 66, 86, 87], CD14 has also been shown to interact with TLR-2 [86, 88], CD36 [89], SR-A [89–91], and CD11b [92, 93], CD14 has been shown to coordinate ligand recognition, signaling and endocytosis of both TLR-4 [66, 73, 94–96] and TLR-2 [97, 98] via lipid raft-mediated and clathrin/dynamin-dependent endocytic mechanisms [99]. Other studies showed that CD36 partially facilitates macropinocytosis of LPS by CD14 while SR-A enhances expression of CD14 and TLR-4 [89]. Here, we found that SP-R210_S_, CD14, and SR-A form a pro-inflammatory complex in SP-R210_L_(DN) cells as revealed by functional assays using individual or a combination of neutralizing antibodies and immuno-precipitation experiments. Furthermore, we show different trafficking mechanisms of CD14 in control and SP-R210_L_(DN) cells in which relocation or secretion of CD14 to the cell membrane after addition of LPS is dynasore-sensitive in control cells but dynasore-insensitive in SP-R210_L_(DN) cells. Initial internalization of CD14 stimulated by LPS is insensitive to dynasore consistent with dynamin-independent internalization. Dynasore, however, partially inhibited LPS-induced TNFα in control cells only, consistent with inhibition of TLR-4 dynamin-dependent endocytosis and full activation of the inflammatory response by endosomal TLR-4 as expected based on previous reports [73]. Induction of TNFαby LPS was also inhibited by the hallmark macropinocytosis inhibitor EIPA in control cells, indicating that macropinocytic internalization of CD14 and/or TLR-4 is needed for downstream activation of the inflammatory response. EIPA and dynasore, however, had no effect on the LPS response in SP-R210_L_(DN) cells, suggesting deployment of a novel inflammatory mechanism when SP-R210_L_ expression is attenuated. Interestingly, inhibition of the small GTPase Rac1 reduced LPS activation in both cells although more effectively in SP-R210_L_(DN) cells, supporting the notion that Rac1 is a common downstream effector of LPS internalization and signaling. Rac1 is one of several GTPases that mediate macropinocytosis [75, 76, 100, 101]. On the other hand, Rac1 may contribute to prolonged activation of NFκB in SP-R210_L_(DN) cells downstream of TLR-4 [102] or SR-A [103]. Taken together, the present findings support the notion that SP-R210_L_ plays a dominant intrinsic role to suppress inflammatory responsiveness of macrophages via a mechanism that modulates CD14 trafficking and cross-talk with the pro-inflammatory isoform SP-R210_S_.

In the lung, SP-R210_L_ is the main isoform in alveolar macrophages. Earlier studies showed that SP-R210_L_ is induced in highly differentiated macrophages whereas SP-R210_S_ is expressed throughout the myeloid lineage [3]. Here, we show that SP-A enhances alveolar macrophage expression of SP-R210_L_ in the local environment. Recent studies showed that alveolar macrophages maintain a pro-inflammatory signature [104–106], indicating that alveolar macrophages are already primed for increased responsiveness to inflammatory agents *in vivo*. Conversely, alveolar macrophages are resistant to tolerogenic effects of LPS and other inflammatory stimuli [105, 107, 108]. Based on the present findings, we propose that SP-R210_L_ controls homeostatic functions of alveolar macrophages while low levels of SP-R210_S_ may be needed to initiate inflammatory responses rapidly.

In the present study we also sought to determine how SP-A affects macrophage responses to LPS in control and SP-R210_L_(DN) cells. We found that treatment of macrophages with SP-A from the same individual prepared side-by-side by either modified butanol/octylglucoside [109] (SP-Am1) or isopropyl ether/butanol/ethanol extraction-based methods [48] (SP-Am2) results in priming [57] or suppression macrophage activation [48] by LPS, confirming earlier results with SP-A obtained by respective methods of purification from different individuals. The differential activities of SP-Am1 and SP-Am2 *in vitro* could be attributed to variable levels of co-isolating proteins that are known to contribute to surfactant function *in vivo* including gp340, a known surfactant protein binding protein [79], the pro-SP-B processing enzyme napsin A [81], and SP-B, which is crucial for surfactant function *in vivo* [53]. In the latter case, multiple studies in side-by-side or separate investigations have shown that Survanta, a clinically used surfactant preparation that contains SP-B and SP-C but not SP-A, exhibits anti-inflammatory activity in studies using mouse and human alveolar macrophages, peripheral blood monocytes, and monocytic cell lines [34, 46, 110–117]. In this regard, increasing studies indicate that SP-B and SP-C and surfactant lipids are not immunologically inert but rather act as antagonists of inflammation [11, 116, 118–120]. However, in an earlier study treatment of mice with SP-A purified by preparative isoelectric focusing had potent inflammatory properties using human monocytic cell lines *in vitro* [41–43, 46] over a number of years, but SP-A prepared in the same manner had anti-inflammatory activity when administered *in vivo* suppressing lung injury in the context of an adaptive immune response to allogeneic bone marrow transplant [121]. Interestingly, in the same injury model, side-by-side comparison with SP-A prepared by a commonly used isobutanol/octylglucoside that had anti-inflammatory properties *in vitro* also had anti-inflammatory activity *in vivo* [121]. In this regard, ligation of SP-R210 with an antibody that recognizes both isoforms resulted in secretion of IL-10 and TGFβ and suppression of lymphocyte proliferation in the context of a secondary immune response to mycobacterial antigen [12]. Taken together, variation in SP-A purity, co-isolating surfactant components, and the biological context in which SP-A is studied are important considerations in understanding SP-A functions *in vivo* and *in vitro*. It is reasonable to consider that co-isolating surfactant proteins contribute to anti-inflammatory activity of SP-A preparations by reducing binding of inflammatory agents to innate immune receptors, although these results may reflect physiological interactions of SP-A and SP-B *in vivo*. These findings highlight that extensive biochemical characterization of SP-A preparations is required to elucidate the interaction of SP-A with macrophage receptors.

**Fig 11:**
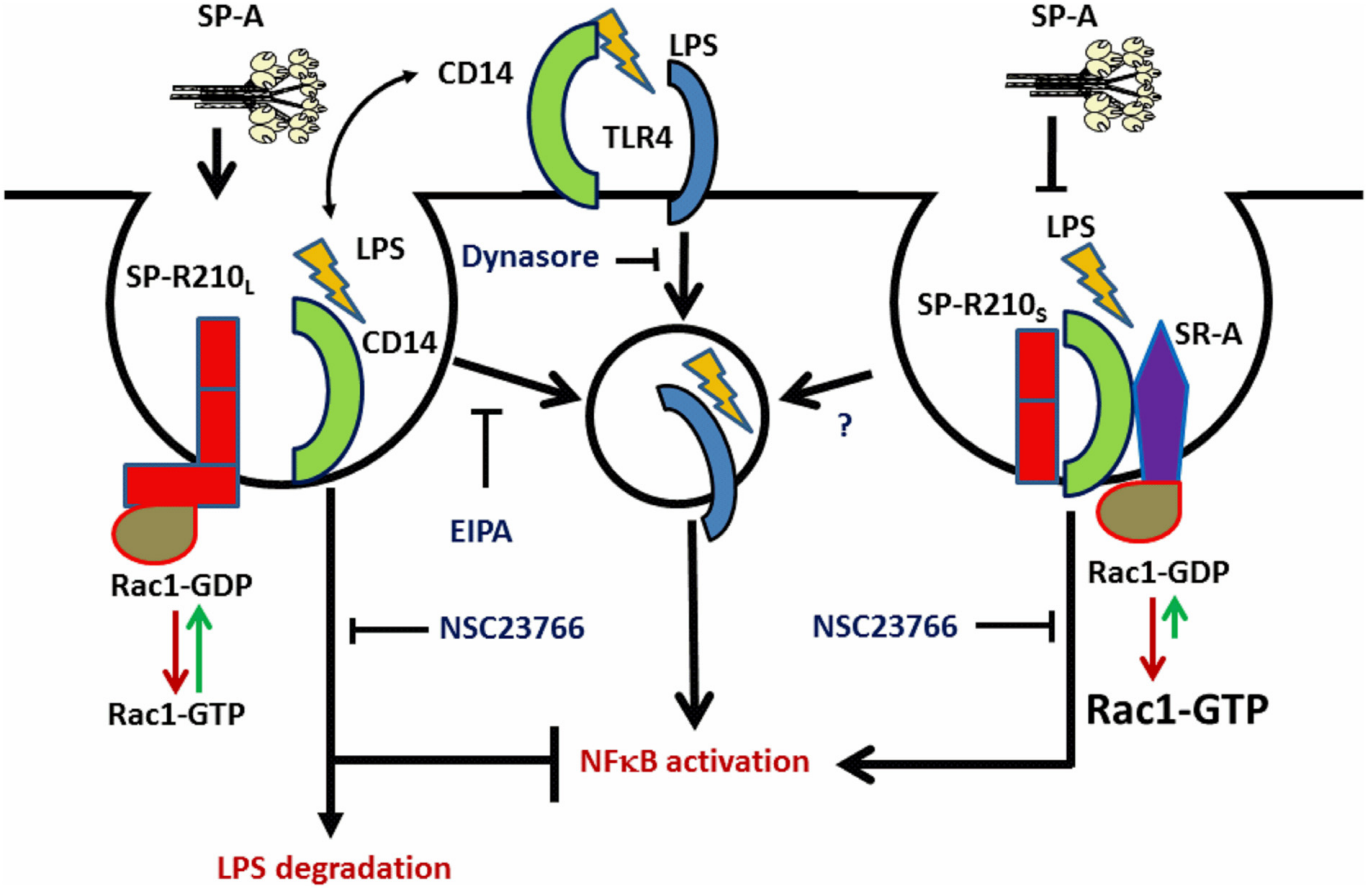
Proposed interaction of SP-R210 isoforms with CD14 and SR-A in macrophage activation. SP-R210_L_ mediates macropinocytosis of LPS-CD14 through interaction with Rac1 resulting in endosomal LPS delivery to TLR-4 and downstream activation of NFκB. Subsequent degradation of LPS results in decrease NFκB signaling. SP-R210_L_-mediated macropinocytosis and signaling are sensitive to both EIPA and NSC23766. TLR-4 signaling from the cell-surface is sensitive to dynasore. Inhibition of SP-R210_L_ expression results in formation of the SP-R210_S_-CD14-SR-A complex. Binding of LPS results in macropinocytosis-like internalization of the SP-R210_S_-CD14-SR-A complex and activation of a feed-forward inflammatory pathway that depends on activation of Rac1 by SR-A. The SP-R210_S_-CD14-SR-A pathway is sensitive to NSC23766 but not EIPA.

Taken together, the present findings support the model depicted on Fig 11. In this model, SP-R210_L_ interacts with the Rac1 signaling pathway to enhance macropinocytosis and clearance of LPS via CD14 with moderate activation of endosomal TLR-4. We propose that the ability of SP-A to maintain high levels of SP-R210_L_ *in vivo* enhances the capacity of alveolar macrophages to clear LPS without overt inflammation, providing a mechanistic explanation for the anti-inflammatory role of SP-A *in vivo*. In turn, SP-R210_L_ modulates expression and localization of innate immune receptors priming macrophages for an appropriate response at increased levels of inflammatory agents in the environment. However, in the absence of SP-R210_L_-mediated regulation, the response to LPS is mediated through a pro-inflammatory complex between SP-R210_S_, CD14, and SR-A in which SR-A is responsible for macropinocytosis-like internalization of LPS and prolonged activation of the rac-1 signaling pathway. In this case, SP-A and possibly other surfactant components may serve to inhibit the SP-R210_S_/CD14/SR-A complex, contributing to regulation and resolution of the inflammatory response. This model is supported by endocytic, biochemical, and functional assays utilizing neutralizing antibodies to SP-R210 and co-receptors and small molecule inhibitors. Domain and isoform specific monoclonal antibodies will help define the expression and function of SP-R210 isoforms in different macrophage populations in the future. SP-R210_L_ is the main isoform on alveolar macrophages [5], indicating that expression of SP-R210_L_ is regulated locally. Our study suggests that differential expression of SP-R210 isoforms determines the inflammatory phenotype of macrophages.

## Supporting Information

S1 Fig SP-A induces expression of SP-R210. Expression of SP-R210 was determined by Western blot analysis in control (A) and SP-R210_L_(DN) (B) cells treated with increasing concentration of SP-A purified by method 1. The cells were also treated with 100 ng/mL LPS. Blots were re-probed with actin as loading control. Control (A) and SP-R210_L_(DN) (B) cells were cultured in 12 well plates for 24 hrs and then treated with increasing concentrations of SP-A, or 100 ng/mL LPS. The band intensity of SP-R210_L_, SP-R210_S_, and actin was determined by densitometry. Densitometry data were normalized to actin and expressed relative to SP-R210_L_ in untreated (NT) control cells (A) and SP-R210_S_ in SP-R210_L_(DN) (B) cells. Data shown are representative of two independent experiments SP-Am1.
(TIF)

S2 Fig SP-A and LPS enhance expression of CD14. Expression of CD14 was determined by Western blot analysis in control (B) and SP-R210_L_(DN) (B) cells treated with increasing concentration of SP-A purified by method 1 or 100 ng/mL LPS for 24 hrs. Blots were re-probed with actin as loading control. The band intensity of CD14 and actin was determined by densitometry. Densitometry data were normalized to actin and expressed relative to CD14 in untreated (NT) cells. Data shown are representative of two independent experiments using SP-Am1.
(TIF)

S3 Fig SP-A preparations enhance or inhibit responsiveness of macrophages to LPS. Control and SP-R210_L_(DN) cells were pretreated with SP-A purified by either method 1 (SP-Am1) or method 2 (SP-Am2) as described in “Materials and methods”. A) Cells were pretreated with 5 or 50 μg/mL SP-Am1 for 24 hrs and then incubated with 100 ng/mL LPS. Levels of secreted TNFα were measured in media by ELISA at 24 (B) hrs after addition of LPS. B) To measure the effect of SP-A purified by different methods, control cells were pre-incubated for 1 hr with 50 μμg/mL SP-Am1 or SP-Am2 and then treated with 100 ng/mL LPS. Secretion of TNFα was measured 4 and 24 hrs after addition of LPS. *p <0.0001; ^#^p<0.04; ^$^p<0.005
(TIF)

S4 Fig Isolation and characterization of SP-A by different methods. SP-A was purified from alveolar proteinosis fluid (APF) obtained by therapeutic lung lavage from different alveolar proteinosis patients using methods 1 and 2 as described in “Materials and Methods”. (A) The purity of SP-A was assessed by silver staining as described by Rabiloud [59]. (B) The presence of SP-B was verified by Western blot analysis. Proteins were separated on reducing (A) or non-reducing (B) 4–17% SDS-PAGE gels. SP-A purified by both methods were free of SP-D (not shown). Arrows indicate positions of SP-B and SP-A. (C) SP-A preparations separated on reducing SDS-PAGE were stained using the Invitrogen SilverQuest kit suitable for mass spectrometry. Gel bands were identified by numbers were subjected to in-trypsin digests and tryptic peptide fingerprints and interrogated by MALDI mass spectrometry. Proteins found in each gel band were identified at 100% confidence index.
(TIF)
