# Supplementary figures and images for: SP-R210 (Myo18A) Isoforms as Intrinsic Modulators of Macrophage Priming and Activation
Source: PLoS One. 2015 May 12;10(5):e0126576. doi: 10.1371/journal.pone.0126576 (PMC4428707; doi:10.1371/journal.pone.0126576)

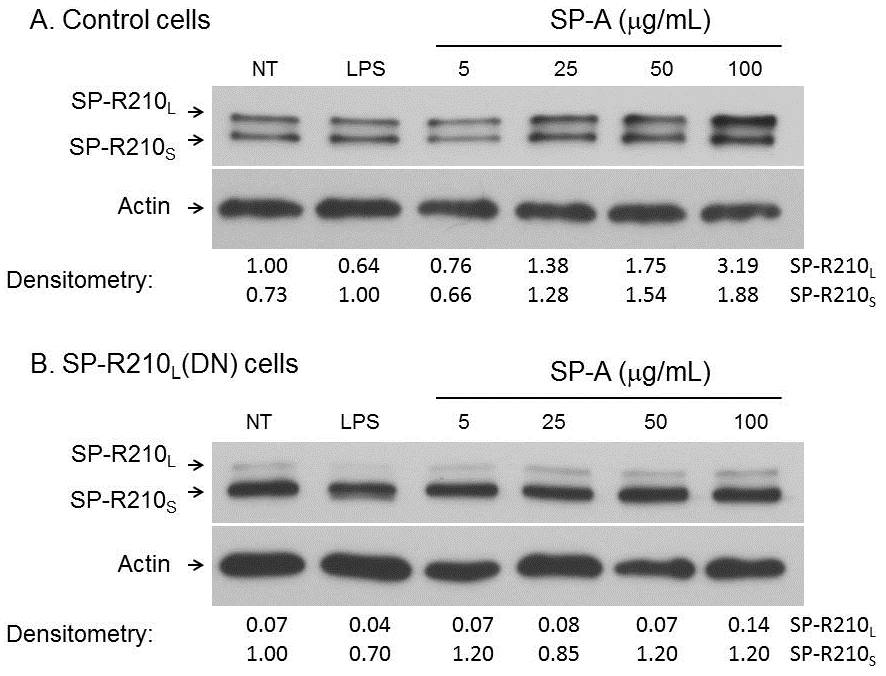

Supplement: S1 Fig — Expression of SP-R210 was determined by Western blot analysis in control (A) and SP-R210L(DN) (B) cells treated with increasing concentration of SP-A purified by method 1. The cells were also treated with 100 ng/mL LPS. Blots were re-probed with actin as loading control. Control (A) and SP-R210L(DN) (B) cells were cultured in 12 well plates for 24 hrs and then treated with increasing concentrations of SP-A, or 100 ng/mL LPS. The band intensity of SP-R210L, SP-R210S, and actin was determined by densitometry. Densitometry data were normalized to actin and expressed relative to SP-R210L in untreated (NT) control cells (A) and SP-R210S in SP-R210L(DN) (B) cells. Data shown are representative of two independent experiments SP-Am1. (TIF) [file pone.0126576.s001.tif]

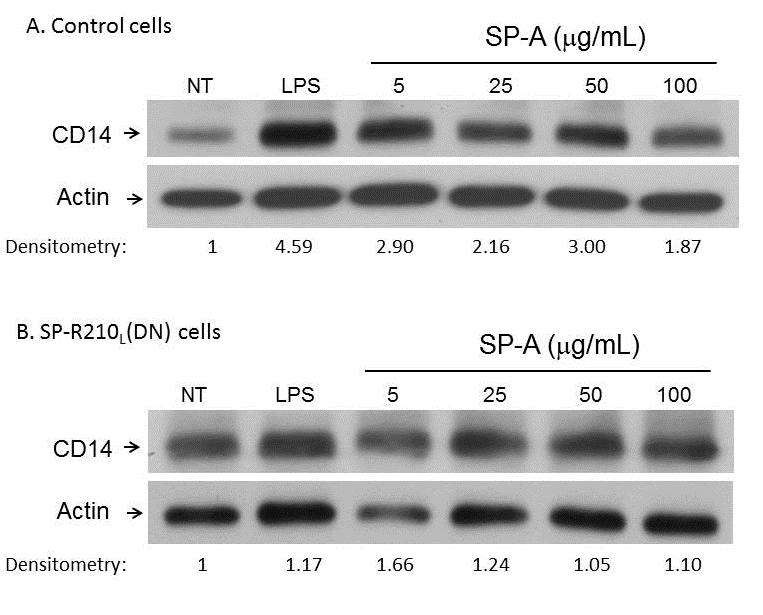

Supplement: S2 Fig — Expression of CD14 was determined by Western blot analysis in control (B) and SP-R210L(DN) (B) cells treated with increasing concentration of SP-A purified by method 1 or 100 ng/mL LPS for 24 hrs. Blots were re-probed with actin as loading control. The band intensity of CD14 and actin was determined by densitometry. Densitometry data were normalized to actin and expressed relative to CD14 in untreated (NT) cells. Data shown are representative of two independent experiments using SP-Am1. (TIF) [file pone.0126576.s002.tif]

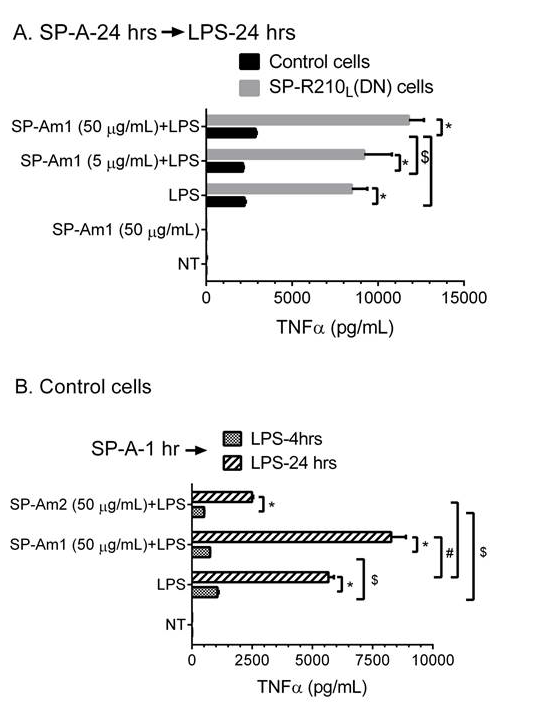

Supplement: S3 Fig — Control and SP-R210L(DN) cells were pretreated with SP-A purified by either method 1 (SP-Am1) or method 2 (SP-Am2) as described in “Materials and methods”. A) Cells were pretreated with 5 or 50 μg/mL SP-Am1 for 24 hrs and then incubated with 100 ng/mL LPS. Levels of secreted TNFα were measured in media by ELISA at 24 (B) hrs after addition of LPS. B) To measure the effect of SP-A purified by different methods, control cells were pre-incubated for 1 hr with 50 μμg/mL SP-Am1 or SP-Am2 and then treated with 100 ng/mL LPS. Secretion of TNFα was measured 4 and 24 hrs after addition of LPS. *p <0.0001; #p<0.04; $p<0.005 (TIF) [file pone.0126576.s003.tif]
